# Non-pharmacological interventions for blood pressure management in hypertensive patients: selection of exercise regimens—a systematic review and network meta-analysis

**DOI:** 10.3389/fendo.2026.1926728

**Published:** 2026-08-03

**Authors:** Renchao Lv, Xinying Wang, Hongfei Li, Zitong Yang, Liaokun Ye, Ming Zhou

**Affiliations:** 1 School of Education, Yunnan Technology and Business University, Kunming, China; 2 School of Physical Education and Sport, Central China Normal University, Wuhan, China; 3 School of Physical Education, Kunming University, Kunming, China

**Keywords:** combined training, exercise training, hypertension, intervention, network meta-analysis

## Abstract

**Objective:**

To compare and rank the effects of exercise modalities on blood pressure in adults with hypertension.

**Methods:**

We conducted a PRISMA-NMA–compliant systematic review and network meta-analysis. PubMed, the Cochrane Library, and Web of Science were searched through May 24, 2026, for randomized controlled trials of exercise interventions. Random-effects models were used, and interventions were ranked using surface under the cumulative ranking curve values. Risk of bias and evidence certainty were assessed with RoB 2, GRADE, and CINeMA.

**Results:**

Thirty trials involving 2,137 participants were included. Compared with standard care, combined training produced the greatest reduction in systolic blood pressure (mean difference [MD], −9.52 mmHg; 95% CI, −13.47 to −5.56) and ranked first. Isometric training and moderate-intensity continuous training also significantly reduced systolic blood pressure. High-intensity continuous training produced the largest reduction in diastolic blood pressure (MD, −7.48 mmHg; 95% CI, −11.54 to −3.43), followed by combined training (MD, −5.84 mmHg; 95% CI, −8.51 to −3.16). No major inconsistency or publication bias was detected.

**Conclusion:**

Combined training may be most effective for lowering systolic blood pressure, whereas high-intensity continuous training may yield the greatest reduction in diastolic blood pressure. Larger, longer-term trials are needed to confirm these findings.

**Systematic review registration:**

https://www.crd.york.ac.uk/prospero/display_record.php?RecordID=1432343, identifier CRD420261432343.

## 1 Introduction

Data released by the World Health Organization (WHO) in 2025 indicate that, in 2024, approximately 1.4 billion adults aged 30–79 years were living with hypertension worldwide, representing about one third of this age group. Nearly 600 million affected individuals were unaware of their condition, and only about 320 million had achieved blood pressure control. WHO identifies hypertension as one of the leading causes of premature mortality worldwide (1). More importantly, the global burden of hypertension is marked by substantial geographic and socioeconomic disparities. Approximately two-thirds of people with hypertension live in low- and middle-income countries, where awareness, treatment, and control rates vary widely and blood pressure control remains below 10% in some settings. The number of adults aged 30–79 years with hypertension increased from approximately 650 million in 1990 to 1.4 billion in 2024, with most of this growth occurring in low- and middle-income countries. These findings underscore that hypertension is not confined to any single country or region, but represents a major public health challenge requiring a coordinated global response (2). Hypertension is defined as persistently elevated blood pressure, commonly a systolic/diastolic pressure of ≥140/90 mmHg. In China, the prevalence of hypertension remains high. Data from the 2018 China Chronic Disease and Risk Factor Surveillance showed that the prevalence of hypertension among adults aged ≥18 years was 27.5%, with higher rates in men than in women and in rural than in urban populations. Among individuals with hypertension, the rates of awareness, treatment, and control were 41.0%, 34.9%, and 11.0%, respectively, indicating that under-recognition, undertreatment, and inadequate control remain major gaps in hypertension management (3).Hypertension can damage the vasculature and major target organs, substantially increasing the risk of cerebral infarction, stroke, heart failure, and renal failure (4).Pharmacological therapy remains the mainstay of hypertension management (5).

However, a substantial body of evidence indicates that structured exercise training and targeted nutritional management can effectively reduce blood pressure (6).Hypertension management requires an integrated approach that combines lifestyle modification with pharmacological therapy. Both the 2023 European Society of Hypertension (ESH) Guidelines and the 2024 European Society of Cardiology (ESC) Guidelines identify regular physical activity and structured exercise training as core components of care and recommend tailoring exercise prescriptions to each patient’s cardiovascular risk, physical capacity, and exercise tolerance (7, 8).Numerous head-to-head randomized controlled trials and meta-analyses have compared the blood pressure-lowering effects of different exercise modalities in individuals with hypertension. The main interventions examined include high-intensity continuous aerobic training (HICT), low-intensity continuous training (LICT), high-intensity interval aerobic training (HIIT), functional fitness training (FFT), isometric handgrip training (IFT), and combined training (CT). However, although current guidelines broadly endorse exercise for blood pressure reduction, clear and consistent evidence is still lacking regarding the comparative efficacy of different exercise modalities and the durability of their antihypertensive effects. Although previous studies generally support the beneficial effects of exercise on blood pressure in this population, their findings remain inconsistent. Moreover, most studies have focused on a single exercise modality, and conventional pairwise meta-analyses are limited to direct comparisons between two exercise interventions. Notably, although several network meta-analyses on this topic have been published in recent years, they differ in eligibility criteria, classification of exercise modalities, outcome assessment, and population definitions. Edwards et al. classified exercise interventions into aerobic training, dynamic resistance training, combined training, high-intensity interval training, and isometric training, with further comparisons across specific exercise formats. Lu et al. primarily categorized aerobic exercise according to training intensity and volume, whereas Gao et al. focused on middle-aged and older adults and adopted broader categories, such as aerobic and static exercise (9–11). These differing classification frameworks may combine interventions with distinct intensities, interval structures, or modes of muscle contraction within the same network node, thereby reducing comparability and complicating clinical interpretation. Previous studies also differed in participant eligibility, baseline blood pressure, outcome definitions, and blood pressure measurement methods. Moreover, potential effect modifiers—including age, exercise intensity, intervention duration, and supervision—have not been examined consistently or adequately in subgroup analyses. These limitations highlight the need for an updated and more granular synthesis of the available evidence.

The available evidence is further constrained by several methodological limitations. Some randomized controlled trials enrolled relatively small samples and used short intervention and follow-up periods, limiting assessment of the durability of blood pressure reduction. Blood pressure was measured using office, home, or ambulatory monitoring, with differences in setting, timing, and measurement procedures across studies. Because ambulatory and office blood pressure capture different aspects of the blood pressure profile, pooling these measures may increase clinical heterogeneity (12). In addition, substantial variation in exercise intensity, session duration, weekly frequency, intervention length, supervision, and adherence may affect the precision of effect estimates and the robustness of intervention rankings. As additional randomized trials become available, an updated synthesis using more detailed and standardized exercise classifications is warranted.

Accordingly, this systematic review and network meta-analysis aimed to compare the effects of different exercise modalities on systolic and diastolic blood pressure in individuals with hypertension and to rank their relative efficacy. The goal was to identify the most effective exercise interventions and provide evidence to guide clinical practice and exercise prescription.

## 2 Materials and methods

This network meta-analysis (NMA) was reported in accordance with the Preferred Reporting Items for Systematic Reviews and Meta-Analyses extension statement for network meta-analyses (PRISMA-NMA; **Supplementary Table 1**) (13). To enhance transparency and methodological rigor, the protocol for this review was prospectively registered with the International Prospective Register of Systematic Reviews (PROSPERO; registration number CRD420261432343).

### 2.1 Data sources and search strategy

PubMed, the Cochrane Library, and Web of Science were systematically searched from database inception to 24 May 2026. The search strategy combined free-text terms and controlled vocabulary related to hypertension, high blood pressure, blood pressure, exercise, physical activity, and therapeutics (**Supplementary Table 2**). No language restrictions were applied.

### 2.2 Selection criteria

1. Population: Eligible participants were patients with hypertension. Following the World Health Organization definition, hypertension was defined as persistently elevated blood pressure, typically ≥140/90 mmHg. Diagnosis was not based on a single reading; rather, it generally required blood pressure measurements on separate days showing systolic blood pressure ≥140 mmHg and/or diastolic blood pressure ≥90 mmHg (14).
2. Randomized controlled trials (RCTs) evaluating any of the following exercise interventions were eligible: high-intensity interval training (HIIT), high-intensity continuous training (HICT), resistance training (RT), moderate-intensity continuous training (MICT), moderate-intensity interval training (MIIT), low-intensity continuous training (LICT), functional fitness training (FFT), isometric training (IFT), and combined training (CT). In this review, CT was defined as a multicomponent exercise programme comprising moderate-intensity aerobic exercise and resistance training.
3. RCTs evaluating comparator interventions, such as standard of care (SOC), were also eligible.
4. The outcomes were systolic blood pressure (SBP) and diastolic blood pressure (DBP).
5. Only randomized controlled trials that assessed blood pressure using 24-hour ambulatory blood pressure monitoring were included.

#### 2.2.1 Exclusion criteria

1. RCTs reporting multiple follow-up time points from the same cohort were excluded.
2. RCTs were excluded if they did not report the outcomes required for this review.
3. Reviews, case reports, retrospective studies, or observational studies.

Before full-text review, records were screened by title and abstract. For each included randomized controlled trial (RCT), two reviewers independently checked all available reports to ensure that the most recently published data were used.

### 2.3 Exercise intensity classification

Exercise interventions were classified according to the primary exercise modality, prescribed intensity, interval structure, and type of muscle contraction. In accordance with the American College of Sports Medicine criteria (15), aerobic exercise intensity was determined primarily using the percentage of maximal heart rate (HRmax), percentage of heart rate reserve (HRR), maximal oxygen uptake (VO_2_max), metabolic equivalents (METs), and rating of perceived exertion (RPE), as follows:

1. Low intensity: 1.5 to <3.0 METs, ≤50% HRmax, <40% HRR, or an RPE of 10–11.
2. Moderate intensity: 3.0 to <6.0 METs, 55%–69% HRmax, 40% to <60% HRR, or an RPE of 12–13.
3. High intensity: ≥6.0 METs, ≥70% HRmax, ≥60% HRR, or an RPE of 14–17.

Exercise intensity was classified according to the main training phase, excluding warm-up and cool-down periods. When a prescribed intensity range spanned two categories, the intervention was classified according to the target intensity maintained during most of the training sessions. Programs with a clearly specified progression toward a higher target intensity were classified according to the final intended intensity. When different intensity indicators yielded inconsistent classifications, two reviewers independently assessed the original study description, prescribed target range, and overall training protocol and reached a consensus.

The exercise intervention nodes were defined as follows:

1. Low-intensity continuous training (LICT): continuous aerobic exercise performed at low intensity without scheduled recovery intervals during the main training phase, such as low-intensity continuous walking or cycling.
2. Moderate-intensity continuous training (MICT): continuous aerobic exercise performed at moderate intensity without scheduled recovery intervals, including brisk walking, treadmill exercise, cycling, and aerobic dance.
3. High-intensity continuous training (HICT): continuous aerobic exercise performed at high intensity without predetermined recovery intervals, such as continuous high-intensity treadmill walking, running, or cycling.
4. Moderate-intensity interval training (MIIT): repeated bouts of moderate-intensity aerobic exercise interspersed with active or passive recovery periods. Programs without a clearly defined recovery phase were not classified as MIIT.
5. High-intensity interval training (HIIT): repeated bouts of high-intensity exercise alternated with active or passive recovery periods, such as four 4-min high-intensity intervals or repeated short bouts of high-intensity cycling or walking.
6. Resistance training (RT): dynamic muscle contractions performed against external resistance, primarily involving concentric and eccentric actions. Eligible interventions included weight machines, free weights, elastic bands, and bodyweight exercises. Purely isometric training and programs with a substantial aerobic component were not assigned to this node.
7. Isometric training (IFT): sustained muscle contractions performed without appreciable changes in joint angle or muscle length, such as isometric handgrip exercise or wall-squat training. Dynamic resistance training was not included in this node.
8. Functional fitness training (FFT): multicomponent exercise primarily intended to improve functional capacity, balance, coordination, flexibility, mobility, or the ability to perform activities of daily living. Interventions such as Tai Chi, yoga, dance, and exergaming were classified as FFT when they could not be clearly assigned to a single continuous aerobic, interval, resistance, or isometric training node.

### 2.4 Data extraction and quality assessment

Data from the eligible randomized controlled trials (RCTs) were independently extracted by reviewers using a standardized extraction form aligned with PRISMA guidance; any discrepancies were resolved through discussion with the second author. Extracted information included first author, publication year, sample size, participant age, sex and geographical distribution, follow-up duration, and the intervention protocols used in the intervention and comparator groups. For continuous outcomes, mean changes from baseline and their corresponding standard deviations (SDs) were extracted whenever available. When studies reported only baseline and endpoint means and SDs, the mean change was calculated as the difference between the two time points, and the SD of the change score was derived from the baseline SD, endpoint SD, and their correlation coefficient (R) using the following formula:


$$SDchange=\sqrt{{\text{Baseline SD}}^{2}+{\text{Endpoint SD}}^{2}-2R\cdot \text{Baseline SD}\cdot \text{Endpoint SD}}$$

When studies reported only baseline and post-intervention means and standard deviations (SDs), the mean change was calculated as the post-intervention mean minus the baseline mean. The SD of the change score was derived from the baseline SD, post-intervention SD, and the assumed correlation between the two measurements. A correlation coefficient of r=0.50r=0.50r=0.50 was applied uniformly to all studies requiring this calculation.

When the correlation coefficient could not be estimated from a given study, R was imputed using the coefficient derived from the trial that reported change scores and had the largest sample size and lowest risk of bias.

Risk of bias was assessed using the Cochrane Risk of Bias 2 (RoB 2) tool. This tool evaluates potential bias across five domains: bias arising from the randomization process, bias due to deviations from the intended interventions, bias due to missing outcome data, bias in measurement of the outcome, and bias in selection of the reported result. Each domain was judged as low risk of bias, some concerns, or high risk of bias (16).

### 2.5 Statistical analysis

Network meta-analyses were performed using Stata/MP 17.0. For a given continuous outcome measured using the same scale and units across studies, effect estimates were expressed as mean differences (MDs) with 95% confidence intervals (CIs). When the same outcome was assessed using different scales or units, standardized mean differences (SMDs) with 95% CIs were used.

Multi-arm trials were expanded into pairwise contrasts using the augment format, while preserving the within-study correlation structure during estimation to avoid artificially small standard errors arising from repeated use of a shared control group. The primary analysis used a random-effects consistency model, with the between-study variance (τ²) estimated by restricted maximum likelihood (REML). When closed loops were present in the network, consistency was assessed using a global inconsistency test, and local inconsistency was examined using the node-splitting approach; a P value <0.10 was considered suggestive of potential inconsistency. Loop-specific inconsistency factors (IFs) were also used to assess agreement within closed loops. If the 95% confidence interval for the IF included 0, this was interpreted as no statistical evidence of disagreement between direct and indirect evidence. Network plots were generated to visualize the geometry of the treatment network.

In the network plots, node size was proportional to the total sample size for each treatment, and edge thickness reflected the number of studies directly comparing the connected interventions. To rank the interventions, we used several ranking metrics, including the surface under the cumulative ranking curve (SUCRA), the probability of being the best treatment (PreBest), and the mean rank, thereby improving the robustness and interpretability of the rankings. Publication bias and small-study effects were assessed using comparison-adjusted funnel plots when more than 10 studies were available. Robustness was examined using leave-one-out sensitivity analyses, in which the random-effects consistency model was refitted after sequentially excluding each study, and the direction and magnitude of the pooled effects were compared. Univariable network meta-regression was further performed to explore the influence of study-level covariates on treatment effects. Regression coefficients, 95% confidence intervals, and Wald test P values were reported; P<0.05 was considered statistical evidence that the covariate modified the treatment effect.

### 2.6 GRADE classification

We assessed the certainty of evidence for the network meta-analysis using CINeMA (Confidence in Network Meta-Analysis) within the GRADE framework. Evidence from randomized controlled trials was initially considered high certainty and then evaluated across six domains: within-study bias, indirectness, imprecision, heterogeneity, incoherence, and between-study bias, including publication bias and small-study effects. Each domain was judged as having no concerns, some concerns, or major concerns. Consistent with GRADE principles, certainty was downgraded by one level for some concerns and by two levels for major concerns, resulting in final ratings of high, moderate, low, or very low certainty.

## 3 Results

### 3.1 Study selection and characteristics

**Figure 1:**
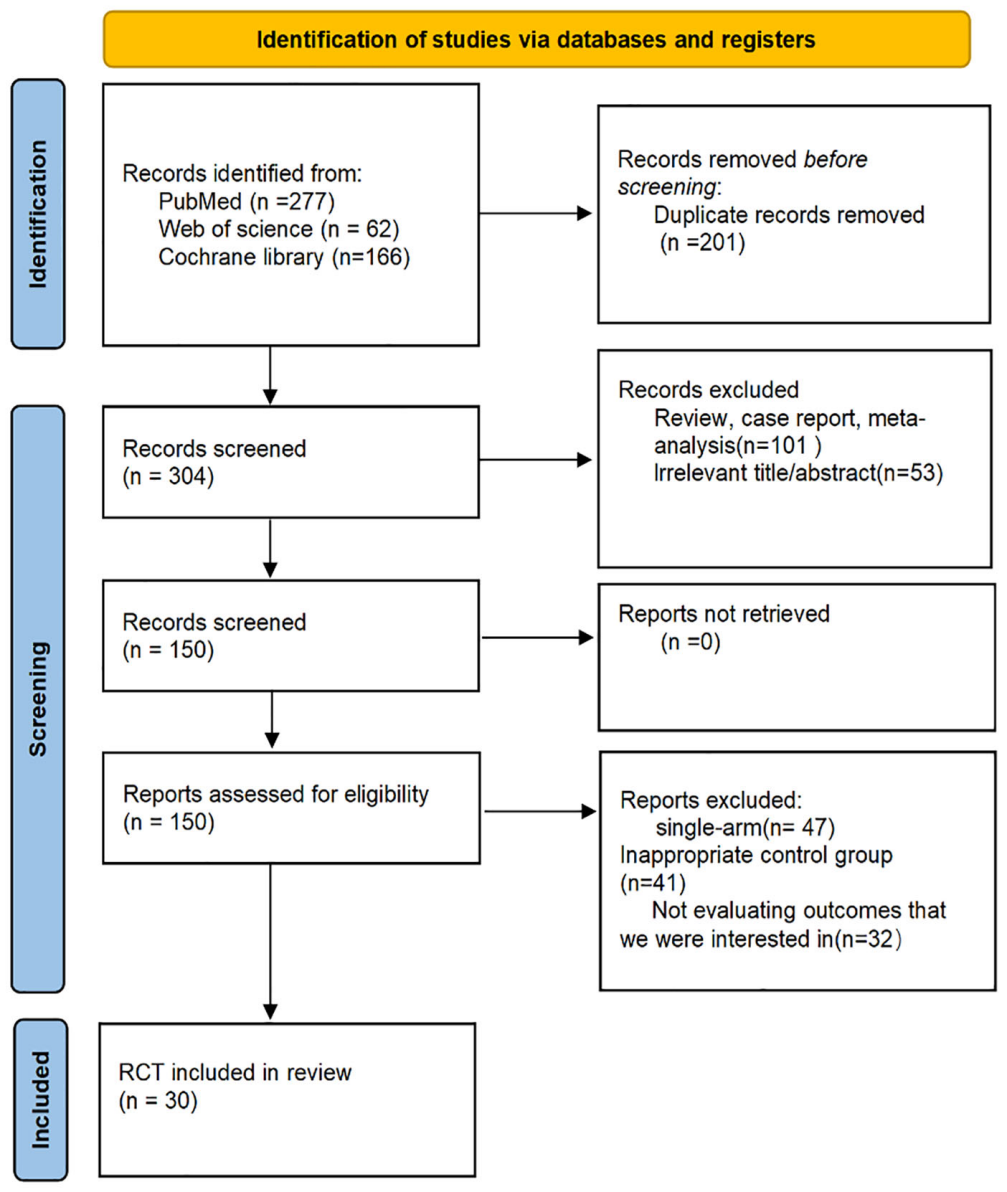
Flow of information the phases of the systematic review and meta-analysis.

The initial database search identified 505 records. After removal of duplicates and exclusion of irrelevant records during title and abstract screening, 150 studies were considered eligible for full-text review. Ultimately, 30 studies met the eligibility criteria and were included in the review (**Figure 1**) (17–46).This review included 2,137 participants assigned across nine exercise intervention categories: high-intensity interval training (HIIT), high-intensity continuous training (HICT), resistance training (RT), moderate-intensity continuous training (MICT), moderate-intensity interval training (MIIT), low-intensity continuous training (LICT), functional fitness training (FFT), isometric training (IFT), and combined training (CT). The included studies were conducted in several countries, including China (n = 3), the United States (n = 5), Brazil (n = 4), and other countries. Study sample sizes were generally moderate. Participants were predominantly middle-aged or older adults, with mean ages clustering between 52 and 68 years, and sex distribution was broadly balanced. Intervention duration varied across studies, most often ranging from 8 to 24 weeks. The characteristics of the included studies are summarized in **Table 1**.

**Table 1:** Characteristics of included trials.

Number	First author and publication year	Age (experimental/control)	Country	Intervention measures in the experimental group	Intervention measures for the control group	Duration (weeks)	Numbers	(n)	Outcome metrics
							intervention	Control	
1	Alemayehu 2023 (17)	45.2	Ethiopia	Aerobic training: Moderate-intensity aerobic dance, initially at approximately 64% of HRmax, gradually increasing to about 76%; Resistance training: Cyclic resistance training, 3 sets × 10 repetitions; Combined training: 30 minutes of resistance training followed by 30 minutes of aerobic training.	usual care	12weeks	11/11/12	12	Body weight, BMI, systolic blood pressure, diastolic blood pressure, resting heart rate, VO_2_max, body fat percentage
2	Banks 2024 (18)	54	America	resistance training: perform exercises such as bench press, hack squat, latissimus dorsi pulldown, leg extension, seated row, leg curl, and plank; the training load is determined based on baseline strength tests and gradually increased by 5%–10% depending on progress.	usual care	9weeks	13	13	Peripheral systolic blood pressure, peripheral diastolic blood pressure, central systolic blood pressure, central diastolic blood pressure, mean arterial pressure, pulse pressure
3	Lopes 2021 (19)	60.1	Portuguesa	Moderate-intensity aerobic exercise training combined with conventional therapy: Each session consists of a 10-minute warm-up, followed by 40 minutes of aerobic exercise performed via cycling and/or walking at an intensity of 50%–70% of VO_2_max (Borg scale 11–14), concluding with a 10-minute relaxation period.	sual care	12weeks	26	27	24-hour ambulatory systolic blood pressure, 24-hour ambulatory diastolic blood pressure, daytime ambulatory systolic blood pressure, daytime ambulatory diastolic blood pressure, nighttime ambulatory blood pressure, heart rate, body composition, cardiopulmonary fitness/VO_2_max
4	Caminiti 2021 (20)	68.3	Italy	Joint Training CT: Each session consists of 10 minutes of warm-up, 40 minutes of aerobic treadmill training, 20 minutes of resistance training, and 10 minutes of relaxation. Resistance training includes leg press, leg extension, shoulder press, chest press, low row, and vertical traction exercises. The resistance intensity is set at 60% of 1RM, with 10 repetitions per set and 2 sets per exercise, followed by a 2-minute rest between sets.	Each aerobic training session consists of a 10-minute warm-up, 60 minutes of treadmill-based aerobic exercise, and 10 minutes of cool-down; exercise intensity is controlled according to the Borg RPE scale level 13–14.	12weeks	28	27	24-hour systolic blood pressure, 24-hour diastolic blood pressure, daytime systolic blood pressure, daytime diastolic blood pressure, nocturnal systolic blood pressure, nocturnal diastolic blood pressure, blood pressure variability (BPV) including mean amplitude variation (MAV) and standard deviation (SD), 24-hour heart rate, daytime heart rate, nocturnal heart rate, exercise test duration, peak exercise heart rate, and body mass index (BMI).
5	Schroeder 2019 (21)	58	America	Moderate-intensity aerobic exercise should not exceed 70% of heart rate; resistance training: 2 sets × 18–20 RM, gradually increasing to 3 sets × 10–14 RM; combined training: 30 minutes of aerobic exercise followed by 30 minutes of resistance training.	sual care	8weeks	17/17/18	17	Peripheral systolic blood pressure, peripheral diastolic blood pressure, central systolic blood pressure, central diastolic blood pressure, resting heart rate, BMI, body weight, waist circumference, lean body mass, fat mass, body fat percentage, VO_2_max, lower limb 1RM, upper limb 1RM, fasting blood glucose, triglycerides, HDL-C, LDL-C, total cholesterol
6	Li 2024 (22)	49.3	China	Tai Chi Training: 24-form Yang-style Tai Chi; each session consists of 10 minutes of warm-up, 40 minutes of core Tai Chi exercises, and 10 minutes of relaxation; at least one group session per week, with an additional three sessions conducted via video-based home practice.	Moderate-intensity aerobic exercise: stair climbing, jogging, brisk walking, cycling; each session consists of 10 minutes of warm-up, 40 minutes of aerobic exercise, and 10 minutes of cool-down; exercise intensity should be monitored.	48weeks	142	141	24-hour ambulatory systolic blood pressure, 24-hour ambulatory diastolic blood pressure, daytime ambulatory systolic blood pressure, daytime ambulatory diastolic blood pressure, nighttime ambulatory systolic blood pressure, nighttime ambulatory diastolic blood pressure, ambulatory pulse rate, blood pressure load, lipid parameters, fasting blood glucose, HbA1c, creatinine, SCORE risk score, waist circumference, body weight, BMI, quality of life (SF-36), adverse events
7	Arija 2018 (23)	68.2	Spain	Supervised walking intervention: Group walking sessions lasting 120 minutes per week, divided into two sessions of 60 minutes each; the walking route covers approximately 5 km and is supervised by nurses and physical activity specialists. Additionally, participants attend monthly socio-cultural activities such as visits to museums, libraries, cultural exhibitions, tourist attractions, and dance classes.	sual care	36weeks	152	55	Cardiovascular disease risk score (CVD risk), systolic blood pressure, diastolic blood pressure, blood pressure control rate, body weight, BMI, smoking status, level of physical activity (IPAQ)
8	Fecchio 2023 (24)	53	Brazil	Dynamic Resistance Training (DRT): 8 resistance exercises; Isometric Hand Strength Training (IHT); Combined Resistance Training (CRT): First perform the same dynamic resistance training as in DRT, followed by the same isometric hand strength training as in IHT.	sual care	10weeks	16/15/15	16	Systolic blood pressure, diastolic blood pressure, mean blood pressure, cardiac output, heart rate, stroke volume, peripheral vascular resistance
9	Sakairi 2025 (25)	56	Japan	YouTube Dance Exercise: Watch approximately 10 minutes of street dance videos and mimic the movements; a total of 5 videos are provided, with an exercise intensity ranging from 4.5 to 7 METs; participants may freely select a video and perform the exercise once daily; no exercise guidance or supervision is provided during the study period.	sual care	8weeks	16	18	Systolic blood pressure (SBP), diastolic blood pressure (DBP), body weight, BMI, muscle mass, body fat percentage, daily time spent on moderate-to-vigorous physical activity (MVPA), adverse events
10	Twerenbold 2023	58	Switzerland	Walking-based High-Intensity Interval Training (HIIT): Each session consists of a 10-minute warm-up at 60%–70% HRmax; followed by 4 sets of 4-minute high-intensity intervals at 80%–95% HRmax; interspersed with 3 minutes of active recovery at 60%–70% HRmax; concluded with a 10-minute cool-down at 60%–70% HRmax, all supervised by exercise scientists.	sual care	8weeks	19	19	Retinal microvascular endothelial function: aFID, vFID, aCON; macrovascular endothelial function: FMD; resting systolic blood pressure, resting diastolic blood pressure, 24-hour systolic blood pressure, 24-hour diastolic blood pressure, post-exercise/post-load systolic and diastolic blood pressure, VO_2_peak, maximum power, body weight, BMI, body fat percentage, visceral fat area, muscle mass, fasting blood glucose, HDL, LDL, triglycerides, hs-CRP.
11	Dimeo 2012 (27)	65.5	Germany	Running treadmill-based walking aerobic interval training; target lactate concentration 2.0 ± 0.5 mmol/L, slightly above the aerobic threshold; existing antihypertensive medications remain unchanged.	sual care	8–12weeks	22	25	Primary outcome: Daily ambulatory systolic blood pressure. Other outcomes: Daily/nightly/24-hour ambulatory systolic and diastolic blood pressure, exercise-induced blood pressure, maximum oxygen uptake, lactate curve, physical performance, arterial compliance (both large and small arteries), cardiac index, body weight, and BMI.
12	Martin 1990 (28)	43.5	America	Aerobic exercise training: brisk walking, jogging, stationary cycling, or a combination thereof; intensity at 65%–80% of maximum heart rate.	sual care	10weeks	10	9	Primary outcome: Diastolic blood pressure (DBP). Other outcomes: Systolic blood pressure (SBP), resting heart rate, exercise capacity/METS, body weight, body fat percentage, 24-hour urinary sodium/potassium excretion.
13	Abrahin 2022 (29)	66.5	Baxi	resistance training: 30–40 minutes per session; aerobic training: power bike exercise; each session includes 5 minutes of warm-up, 30 minutes of continuous moderate-intensity exercise, and 5 minutes of cool-down; the primary training intensity ranges from 60% to 75% of HRmax.	sual care	38weeks	20/20	21	Primary/core outcomes: SBP, DBP; other outcomes: resting heart rate, double product, fasting blood glucose, HbA1c, total cholesterol, HDL, LDL, triglycerides, TG/HDL ratio, functional performance indicators including TUG, grip strength, 30-second arm curl, and 30-second sit-to-standing test.
14	Herawati 2025 (30)	59.6	Indonesia	High-intensity self-weight interval training	sual care	10weeks	20	24	SBP, DBP, resting heart rate (HR), 6-minute walk test (6MWT), grip strength, total cholesterol, LDL, HDL, triglycerides
15	López-Ruiz 2025 (31)	54.4	Spain	Pure aerobic endurance training: The primary session consists of 40 minutes of continuous endurance cycling at an intensity of 55%–70% HRR, followed by 10 minutes of relaxation and static stretching; Combined training: strength training combined with continuous endurance training based on individual load-speed relationship assessments.	sual care	12weeks	25/25	25	systolic blood pressure (SBP), diastolic blood pressure (DBP), mean arterial pressure (MAP), pulse pressure (PP), heart rate-blood pressure product (RPP), resting heart rate (RHR); body composition parameters: BMI, lean mass, body fat percentage, visceral fat percentage, waist circumference; metabolic parameters: total cholesterol, HDL, LDL, triglycerides, blood glucose; physical fitness indicators: grip strength, 1RM for upper and lower limbs, mean propulsive velocity (MPV), VO_2_peak, power output (WATT).
16	Lim 2025 (32)	53.7	Korea	DASH dietary education/advisory and monitoring services, along with regular aerobic exercise counseling; each session includes 10 minutes of warm-up, 40 minutes of aerobic exercise, and 10 minutes of cool-down.	sual care	12weeks	35	35	Primary Outcome: Change in 24-hour Ambulatory Systolic Blood Pressure
17	Pinto 2025 (33)	71.3	Portuguesa	Home-based isometric grip strength training (IFT): Perform 4 sets of bilateral grip contractions, each lasting 45 seconds at 50% of the maximum voluntary contraction force, with a 1-minute rest between sets; Home-based aerobic exercise training: Moderate-to-high-intensity walking for 30 minutes at an intensity of 50%–70% of estimated VO_2_max.	sual care	8weeks	27/26	23	Primary outcome: 24-hour dynamic systolic blood pressure changes; Secondary outcomes: Other dynamic blood pressure parameters, including SBP, DBP, MAP, and PP; Additionally, intervention adherence and adverse events were recorded.
18	Taylor 2018 (34)	43.8	Britain	Home-based isometric exercise training (IET): unsupervised wall squats performed at individualized knee angles, with heart rate monitoring during training.	sual care	11weeks	24	24	24-hour ambulatory blood pressure, daytime/nighttime ambulatory blood pressure, blood pressure variability, cardiac autonomic regulation, hemodynamic parameters, inflammatory markers, and vascular biomarkers
19	Izadi 2017 (35)	61.7	Iran	High-Intensity Interval Training (HIIT): Power bike exercise; 10 sets of 1.5-minute high-intensity intervals at 85%–90% of the maximum heart rate (HRR); active recovery periods of 2 minutes at 50%–55% HRR between each set	sual care	6weeks	15	15	Blood pressure parameters: resting SBP and DBP; Exercise capacity/hydrocardiac parameters: VO_2_peak, maximum heart rate, resting heart rate, post-exercise heart rate recovery at 1/2/3 minutes, exercise test duration; Blood biomarkers: plasma apelin, NOx, ET-1.
20	Hou 2023 (36)	67.9	China	Exergames: Motion-sensing sports games like Nintendo Wii Sports, featuring tennis, bowling, baseball, and golf; simulate physical activities using the Wii controller. Cycling training: horizontal cycling with adjustable cadence or resistance based on heart rate to maintain moderate exercise intensity.	sual care	16weeks	36/38	38	Primary outcomes: SBP and DBP. Secondary outcomes: executive functions, including working memory (N-Back task), cognitive flexibility (Trail Making Test), and inhibitory control (Stroop test); additional measures included physical activity, dietary intake, heart rate, and gaming performance.
21	Alzahrani 2023 (37)	74.4	Saudi Arabia	Low-to-moderate intensity aerobic exercise training: fixed-power bicycle/dyndamic bike; Week 1 starts at 20 minutes at 30% heart rate, gradually progressing to 45 minutes at 50% heart rate; each session includes 10 minutes of warm-up and cool-down; training is supervised by a physical therapist with intensity controlled via heart rate monitoring.	sual care	8weeks	12	12	Primary outcomes: resting systolic blood pressure (SBP) and diastolic blood pressure (DBP). Secondary outcomes: resting heart rate, BMI/body fat percentage, total cholesterol, LDL, HDL, and Katz Functional Index for Daily Living.
22	Gambassi 2024 (38)	70.3	Baxi	Elastic resistance-based dynamic power training combined with endurance training: Perform strength/power exercises using Thera-Band elastic bands, including vertical chest press, plantar flexion, seated rowing, hip abduction, elbow flexion, and trunk flexion, alternating with seated standing/chair squats; complete the concentric phase as quickly as possible, with an eccentric phase lasting approximately 3 seconds; intensity is controlled using RPE, progressing from low intensity (approximately 2 points) to moderate intensity (approximately 3 points). Training progression: First 3 weeks – 2 sets × 6 repetitions + approximately 12 minutes of walking; Middle 3 weeks – 2 sets × 8 repetitions + approximately 15 minutes of walking; Last 2 weeks – 2 sets × 10 repetitions + 18 minutes of walking.	sual care	8weeks	13	13	Arterial stiffness parameters: pulse pressure (PP), central pulse pressure, pulse wave velocity (PWV); hemodynamic parameters: systolic blood pressure (SBP), diastolic blood pressure (DBP), central SBP, central DBP
23	Nemoto 2021 (39)	61.7	Japan	Isometric grip strength training: Use a spring grip strength trainer at home or workplace; apply a force approximately 30% of the individual’s maximum voluntary grip strength; perform 4 sets of 2-minute isometric contractions per session, with 1-minute rest between sets; perform in a standing position; continue for 8 weeks.	sual care	8weeks	53	53	Primary Outcome: Change in 24-hour Ambulatory Systolic Blood Pressure
24	Ma 2018 (40)	69	China	Group 24-style Simplified Tai Chi and routine hypertension care. The Tai Chi session consists of a 10-minute warm-up, 70 minutes of instruction and practice, followed by a 10-minute relaxation period; participants are then grouped according to residential areas for group exercises in parks or similar venues, with instructors providing guidance twice monthly.	sual care	24weeks	55	58	SBP, DBP, BMI, waist circumference; social support; depression symptoms measured by CES-D; health-related quality of life assessed with SF-36, including physical and mental health dimensions
25	Chauhan 2017 (41)	52.3	India	Yoga Practice: Following the government-approved Indian yoga regimen, which includes prayer, warm-up/joint mobilization, asanas (postures), Kapalbhati breathing exercises, pranayama (breathing regulation), meditation (Dhyana), Sankalpa (intention cultivation), and Shanti Patha (path to peace); asanas encompass standing, seated, prone, and supine positions.	Usual care	4weeks	64	26	BMI、收缩压SBP、.舒压DBP
26	Dhungana 2021 (42)	47.8	Nepal	A health worker-led structured yoga intervention comprising: a 5-day centralized yoga training session, 2 hours of health education, and 90 days of home-based yoga practice; the yoga program includes postures, breathing exercises, and meditation/re relaxation techniques.	sual care	12weeks	61	60	Primary outcome: Systolic blood pressure (SBP) at 90-day follow-up; secondary outcomes: diastolic blood pressure (DBP), body mass index (BMI), and resting heart rate; adverse events were also recorded.
27	Georgiades 2000 (43)	47.8	America	Aerobic exercise training: supervised exercise; intensity at 70%–85% of the initial heart rate reserve; each session includes 10 minutes of warm-up, 45 minutes of cycling and walking (gradually progressing to jogging), followed by 10 minutes of cool-down; maintenance of previous diet is required; combined with aerobic exercise training and behavioral weight-loss interventions; weekly group sessions for 30 minutes over 26 weeks; goal is a weekly weight loss of 1–2 pounds achieved through lifestyle modifications to reduce total caloric and fat intake.	sual care	24weeks	36/44	19	Measured parameters included systolic blood pressure (SBP), diastolic blood pressure (DBP), heart rate (HR), cardiac output (CO), stroke volume (SV), and total peripheral resistance (TPR) under resting and mental stress conditions. Mental stress tasks comprised public speaking, anger recall interviews, mirror drawing, and cold stress test.peak oxygen consumption (VO_2_max), body weight, and body mass index (BMI).
28	Hagberg 1989	64	America	Moderate-intensity aerobic training: primarily walking; conducted under supervision for the first month, followed by home-based walking; target intensity approximately 50% of VO_2_max, with an actual average of about 53% VO_2_max; each session lasting approximately 1 hour, performed three times per week. High-intensity aerobic training: target intensity 70%–85% of VO_2_max, with an actual average of about 73% VO_2_max.	sual care	37weeks	11/10	9	SBP, DBP, mean blood pressure (MBP); VO_2_max; resting hemodynamics: cardiac output (CO), heart rate (HR), stroke volume (SV), total peripheral resistance (TPR); body weight, body fat percentage; plasma renin activity, norepinephrine, epinephrine, insulin; urinary sodium excretion, blood volume
29	Cahu Rodrigues 2019 (45)	61	Baxi	Isometric grip strength training: Perform 4 sets of 2-minute isometric grip contractions alternately with both hands; intensity at 30% of maximal voluntary contraction (MVC); rest for 1 minute between sets; adjust the load in week 6. The original randomized controlled trial (RCT) included home-based IHT and supervised IHT; in this study, participants with blood pressure responses were pooled into a single IHT group for analysis.	sual care	12weeks	17	16	Main blood pressure parameters: SBP, DBP. Vascular function/structure indicators: central pulse wave velocity (cPWV), peripheral pulse wave velocity (pPWV), flow-mediated dilation of the brachial artery (FMD), and shear rate area under the curve (AUC). Oxidative stress/inflammation markers: AOPP, MDA, total thiols, IL-1β, IL-6, IL-10, TNF-α, CRP.
30	Pagonas 2017 (46)	60.5	Germany	Isometric grip strength training: Use the Zona device; perform 2 sets of 2-minute contractions per hand at 30% of maximum grip strength, with a 1-minute rest between each contraction; 5 times per week for 12 weeks. Aerobic exercise training: Moderate-intensity endurance exercises, 30 minutes per session, 3–5 times per week.	sual care	12weeks	24/19	23	Primary outcome: 24-hour ambulatory systolic blood pressure (SBP). Secondary outcomes: 24-hour ambulatory diastolic blood pressure (DBP); daytime/nighttime ambulatory SBP and DBP; pulse wave velocity (PWV); systemic vascular resistance (SVR); large artery elasticity index (C1); small artery elasticity index (C2).

### 3.2 Risk-of-bias assessment

**Figure 2:**
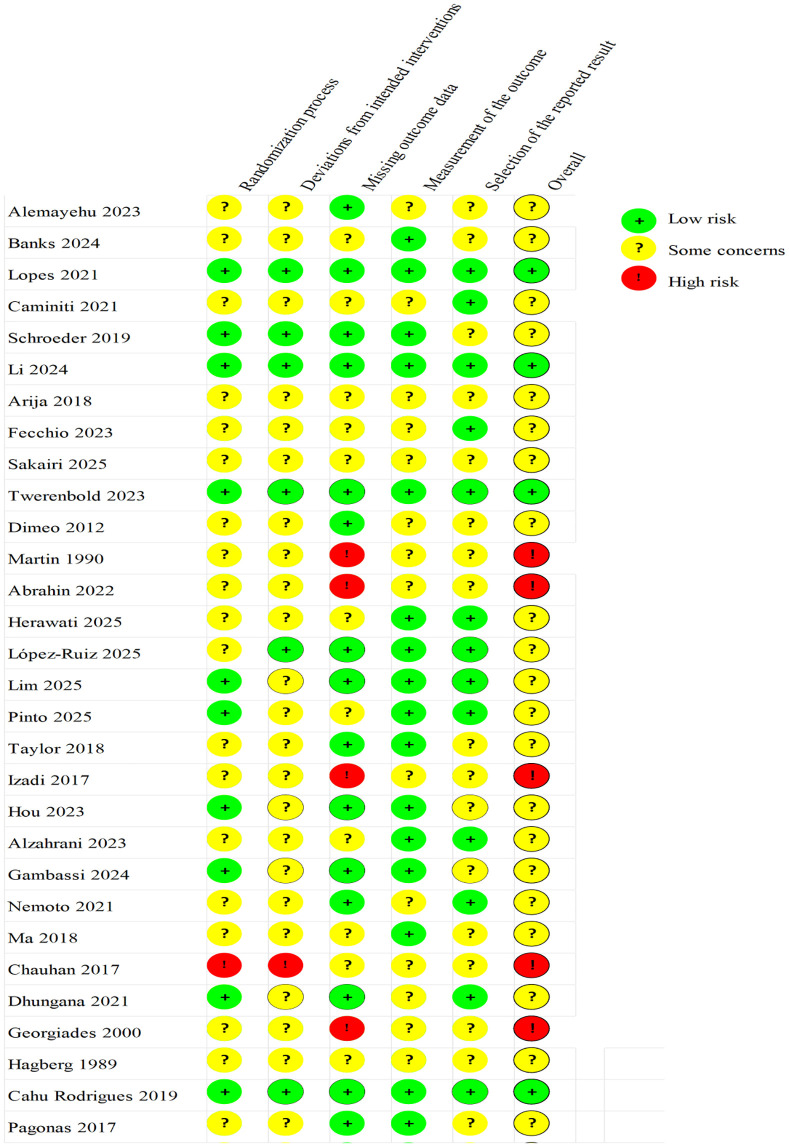
Literature quality assessment of ROB2.

Risk of bias was assessed for the 30 included randomized controlled trials using RoB 2 (**Figure 2**). Risk of bias was assessed across five domains: (1) bias arising from the randomization process; (2) bias due to deviations from the intended interventions; (3) bias due to missing outcome data; (4) bias in outcome measurement; and (5) bias in the selection of the reported result. Based on the signaling questions and the RoB 2 decision algorithms, each domain and the overall risk of bias were rated as “low risk,” “some concerns,” or “high risk.” Two reviewers performed the assessments independently. Disagreements were resolved through discussion, with consultation of a third reviewer when necessary. Overall, 4 studies were judged to be at low risk of bias, 21 raised some concerns, and 5 were rated as high risk, indicating a mixed risk-of-bias profile. Low-risk judgments were assigned to 10 studies for the randomization process, 6 for deviations from the intended interventions, 15 for missing outcome data, 16 for outcome measurement, and 13 for selection of the reported result.

### 3.3 Consistency assessment

**Figure 3:**
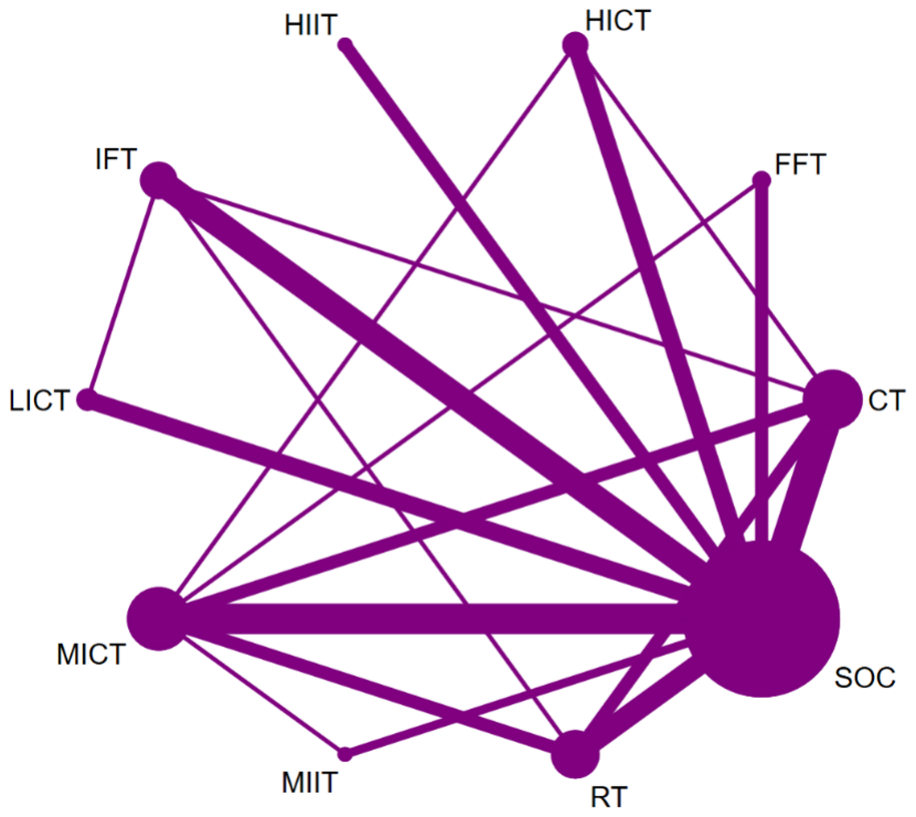
Network diagram illustrating the associations between different exercise training modalities and systolic blood pressure in hypertensive patients.

**Figure 4:**
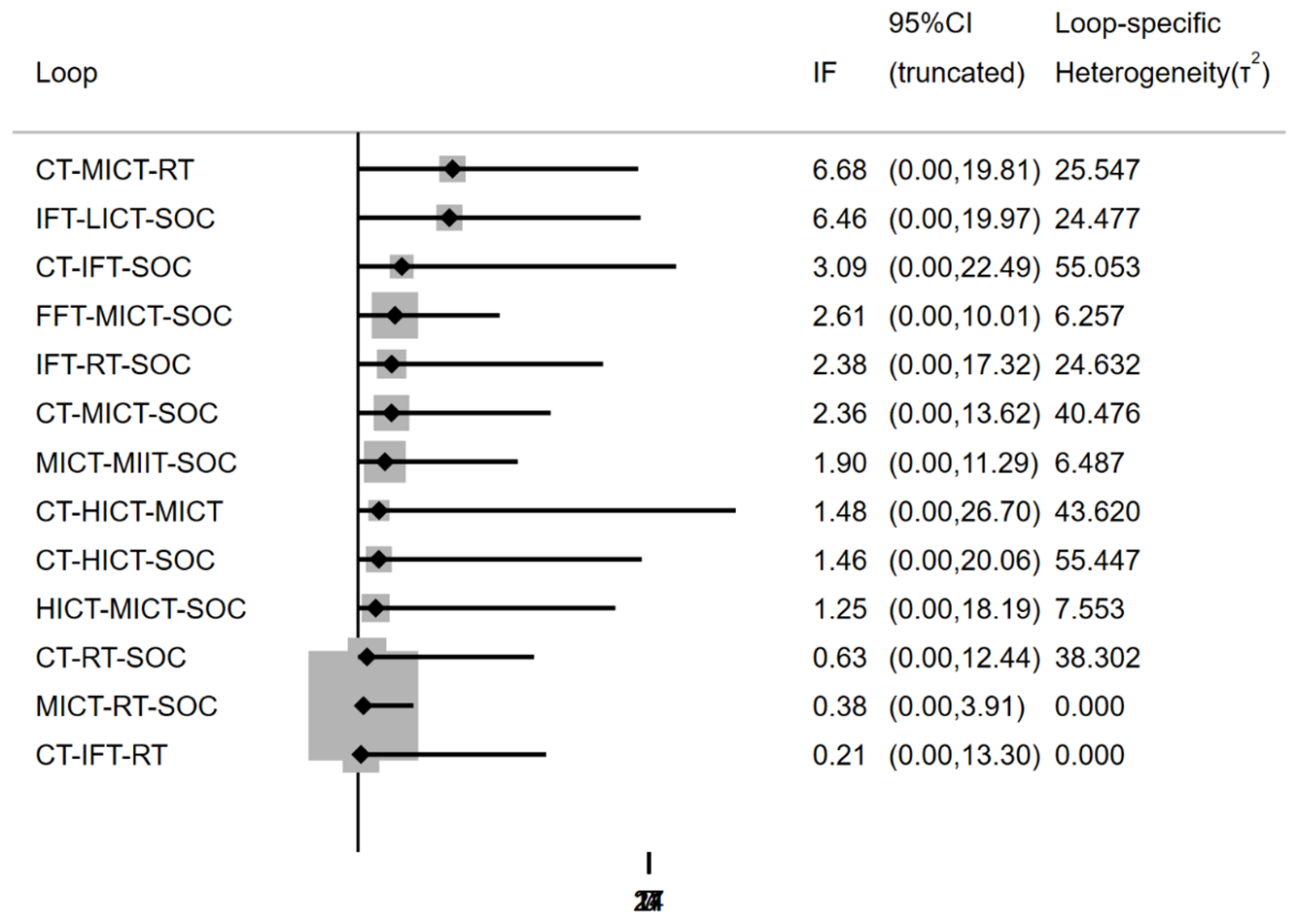
Analysis of inconsistency in the SBP cycle for outcome indicators.

**Figure 5:**
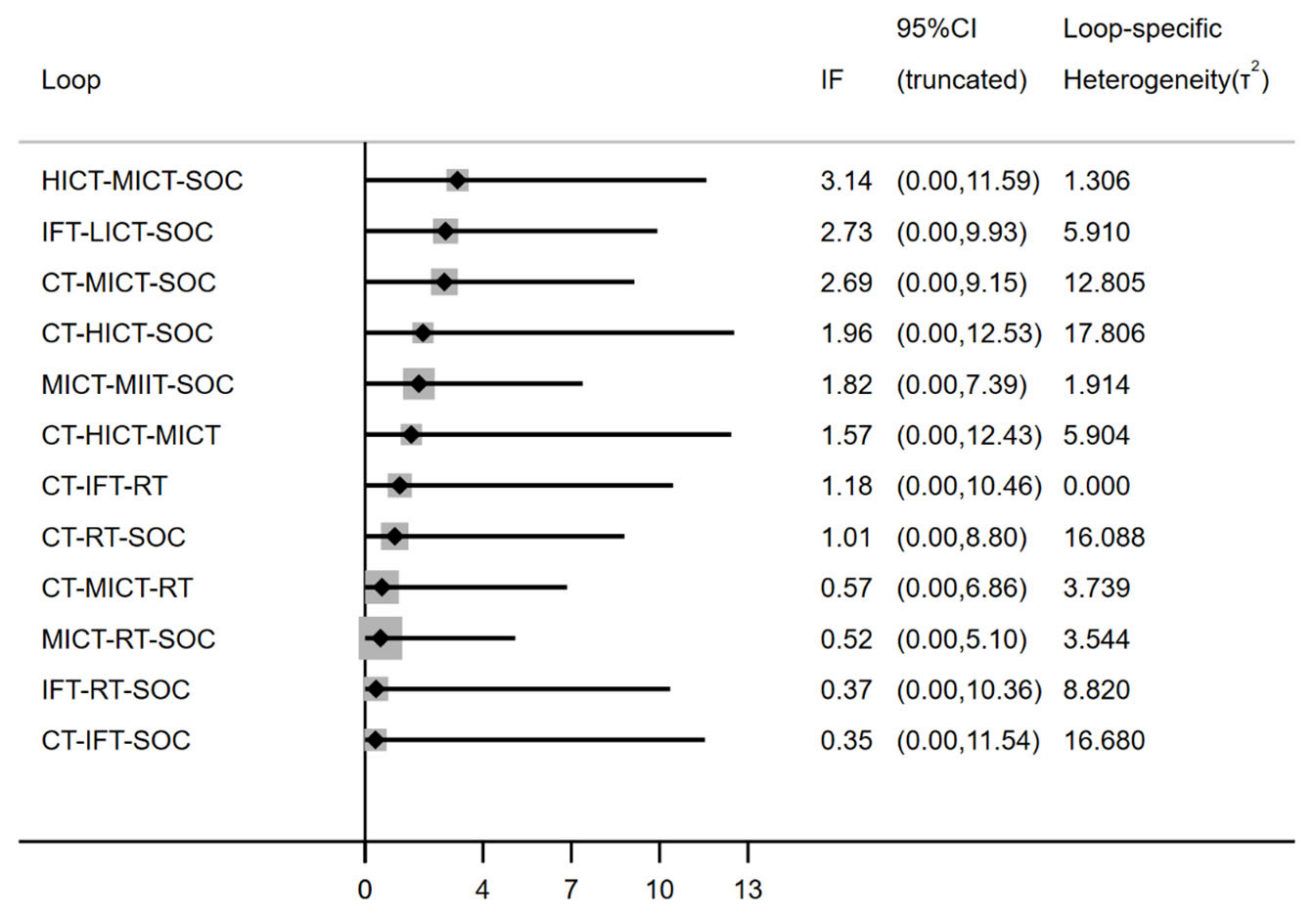
Analysis of circular inconsistency in outcome measures (DBP).

Closed loops were present in the treatment networks for both SBP and DBP (**Figure 3**). Global inconsistency tests showed P values >0.05 for both outcomes, indicating no statistically significant global inconsistency (**Supplementary Table 3**). Local inconsistency was further assessed using the node-splitting approach, and all P values for the node-splitting comparisons were also >0.05 (**Supplementary Tables 4**, **S5**). In addition, loop-specific inconsistency analyses were performed to examine agreement between direct and indirect evidence. The 95% confidence intervals for all inconsistency factors crossed 0, suggesting good overall consistency of the network models (**Figures 4** and **5**). Accordingly, the primary network meta-analyses were conducted using consistency models.

### 3.4 SBP

**Figure 6:**
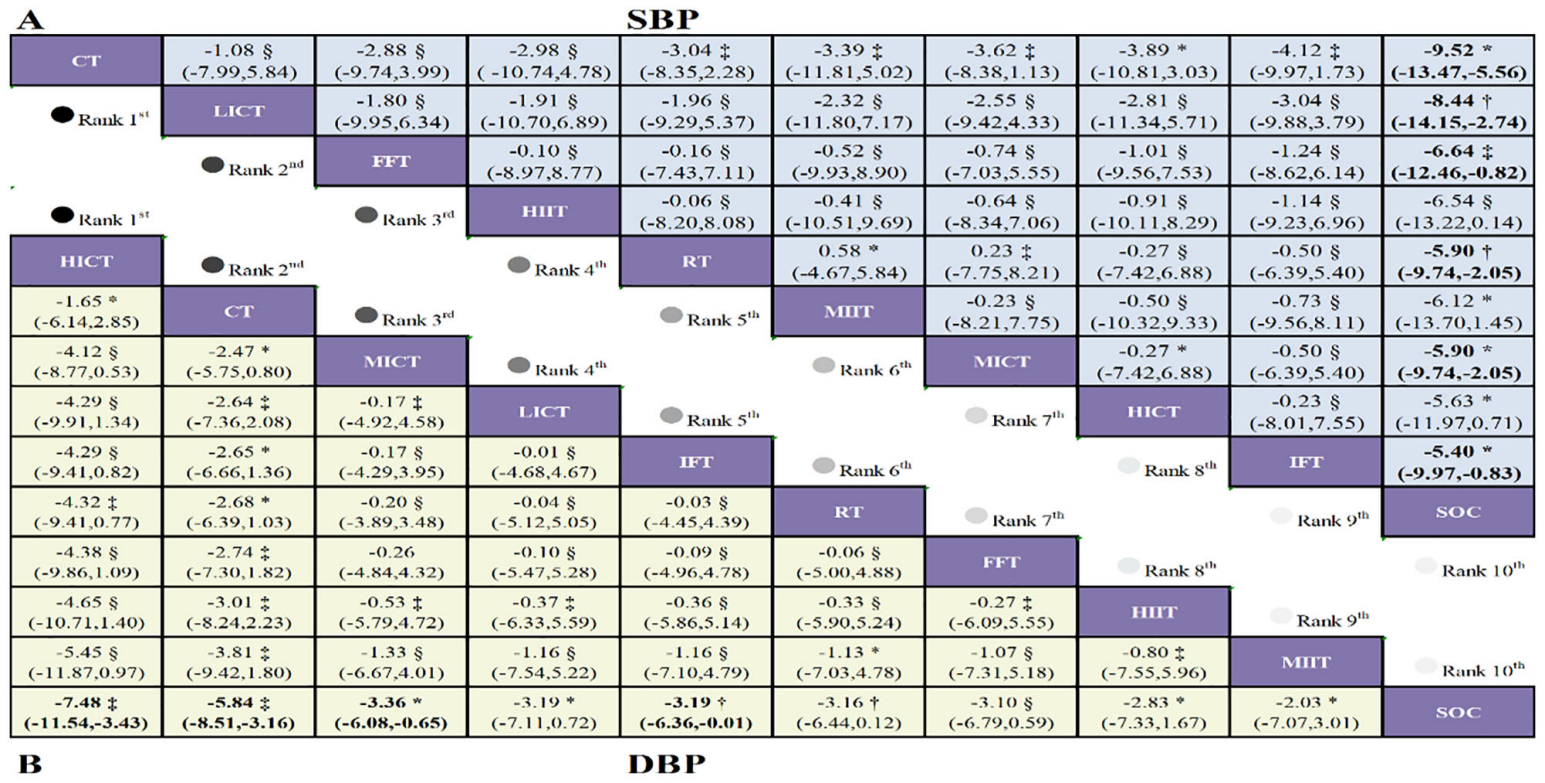
LComparative efficacy plot of exercise interventions for patients with hypertension. Panel **(A)** represents SBP, and Panel **(B)** represents DBP. The treatment effects for DBP are presented in the lower-left triangular area (yellow shaded), and the effects for SBP are shown in the upper-right triangular area (blue shaded). The certainty of evidence is graded as follows: *High certainty, ^†^Moderate certainty, ^‡^ Low certainty, ^§^ Very low certainty.

For SBP, data were available from 30 studies of 10 interventions involving 2,137 patients with hypertension. Compared with SOC, high-certainty evidence supported a significant reduction in SBP with combined training (CT; MD = −9.52 mmHg, 95% CI −13.47 to −5.56). Significant reductions were also observed with isometric handgrip training (IFT; MD = −5.40 mmHg, 95% CI −9.97 to −0.83) and moderate-intensity continuous training (MICT; MD = −5.90 mmHg, 95% CI −9.74 to −2.05; **Figure 6A**). For comparisons that did not reach statistical significance, high-certainty evidence showed that the point estimates favored moderate-intensity interval training (MIIT) (MD −6.12 mmHg, 95% CI −13.70 to 1.45) and high-intensity continuous training (HICT) (MD −5.63 mmHg, 95% CI −11.97 to 0.71) over SOC. However, because the corresponding 95% confidence intervals included the null value, the available evidence did not establish a significant reduction in SBP. These findings should not be interpreted as evidence that MIIT or HICT is equivalent to SOC. The direct evidence comprised 7 studies involving 284 participants for CT versus SOC, 7 studies involving 297 participants for MICT versus SOC, and 6 studies involving 315 participants for IFT versus SOC. These study and participant counts refer specifically to the direct head-to-head evidence, whereas the reported effect estimates and 95% confidence intervals are network estimates incorporating both direct and indirect evidence.

**Figure 7:**
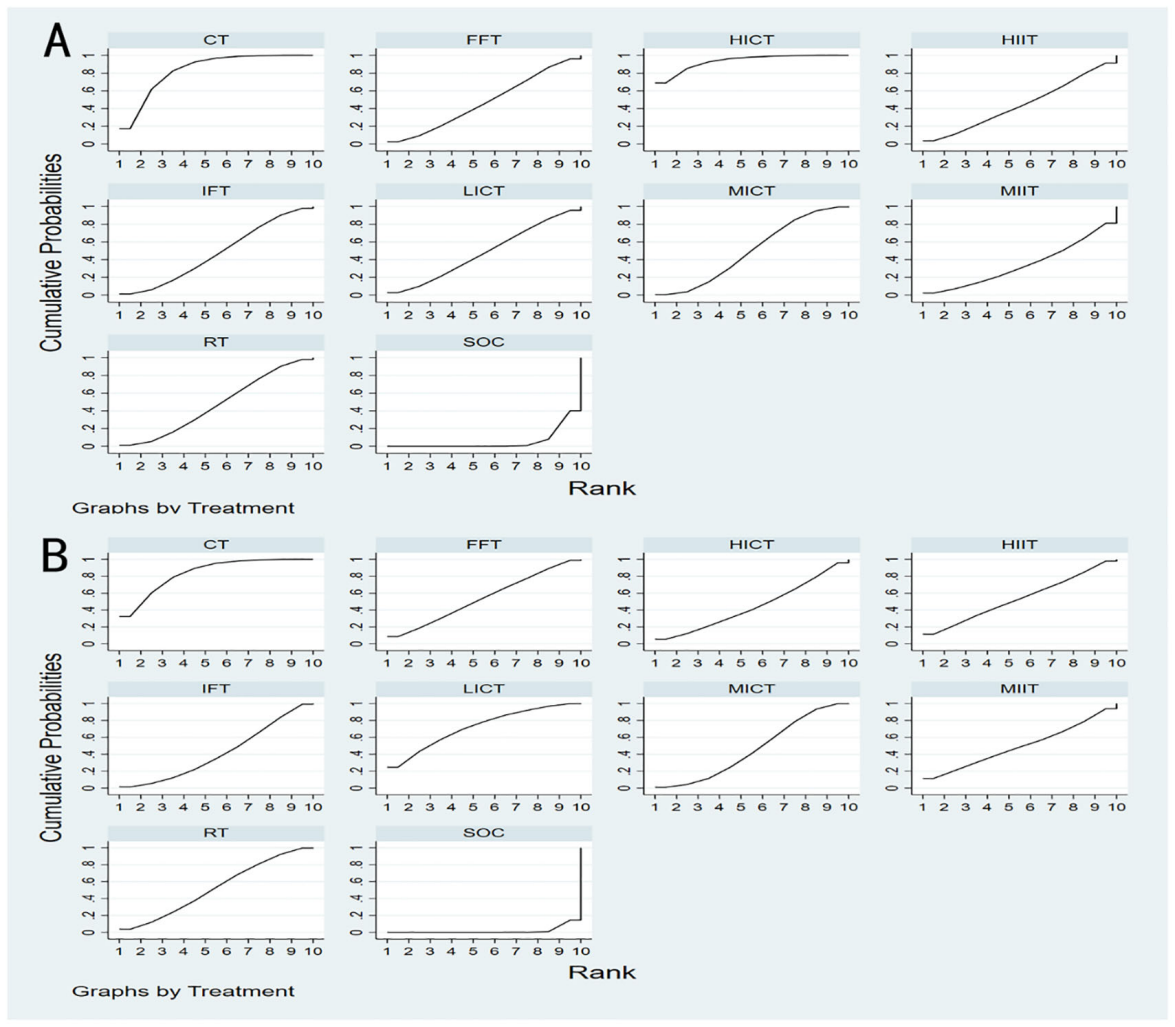
Outcome parameter RANK, where **(A)** represents DBP and **(B)** represents SBP.

Based on the SUCRA rankings, combined training (CT) ranked highest (83.8%), followed by low-intensity continuous training (LICT; 72.1%), indicating that these two interventions were among the more favorable options overall. The probability analysis showed that CT had a 32.5% probability of being the best-performing intervention, compared with 24.7% for LICT (**Figure 7B**; **Supplementary Table 6**). However, SUCRA values and probabilities of being ranked best reflect only the relative ordering of interventions and do not establish statistically significant or clinically meaningful differences between them. These rankings should therefore be interpreted alongside the effect estimates, confidence intervals, and certainty of evidence.

### 3.5 DBP

For DBP, the analysis included 30 studies evaluating 10 interventions in 2,137 patients with hypertension. Low-certainty evidence indicated that high-intensity continuous training (HICT) significantly reduced DBP compared with standard care (SOC) (MD −7.48 mmHg, 95% CI −11.54 to −3.43). A significant reduction was also observed with combined training (CT) (MD −5.84 mmHg, 95% CI −8.51 to −3.16) (**Figure 6B**).

Among comparisons that did not reach statistical significance, moderate-certainty evidence suggested that resistance training (RT) favored a reduction in DBP relative to SOC (MD −3.16 mmHg, 95% CI −6.44 to 0.12), while high-certainty evidence similarly favored high-intensity interval training (HIIT) (MD −2.83 mmHg, 95% CI −7.33 to 1.67). By contrast, the certainty of evidence for functional fitness training (FFT) versus SOC was very low (MD −3.10 mmHg, 95% CI −6.79 to 0.59). Because all corresponding 95% confidence intervals included the null value, the available evidence was insufficient to establish significant reductions in DBP. These findings should not be interpreted as evidence that these interventions are equivalent to SOC.

The direct evidence comprised 4 studies involving 127 participants for HICT versus SOC and 7 studies involving 284 participants for CT versus SOC.

Based on SUCRA values, high-intensity continuous training (HICT) ranked first (93.4%), followed by combined training (CT) (83.3%), suggesting that these interventions had the most favorable overall efficacy profiles. The probability rankings were concordant: HICT had the highest probability of being the best-performing intervention (68.8%), followed by CT (17.2%) (**Figure 7A**; **Supplementary Table 7**).

### 3.6 Meta-analysis, sensitivity analysis, and publication

BiasKey baseline characteristics were generally balanced across treatment groups, with no important imbalances observed. Meta-regression analyses showed that neither mean age nor intervention duration significantly influenced treatment effects, supporting the plausibility of the transitivity assumption (**Supplementary Tables 8**, **S9**).

Leave-one-out sensitivity analyses were conducted to assess the influence of individual studies on the network effect estimates. The results showed that, regardless of which study was omitted, the direction of the relative effects for each exercise intervention versus SOC remained unchanged. Effect estimates varied only modestly, 95% confidence intervals largely overlapped, and conclusions regarding statistical significance were not materially altered, indicating that the primary findings were robust (**Supplementary Material 10**).

**Figure 8:**
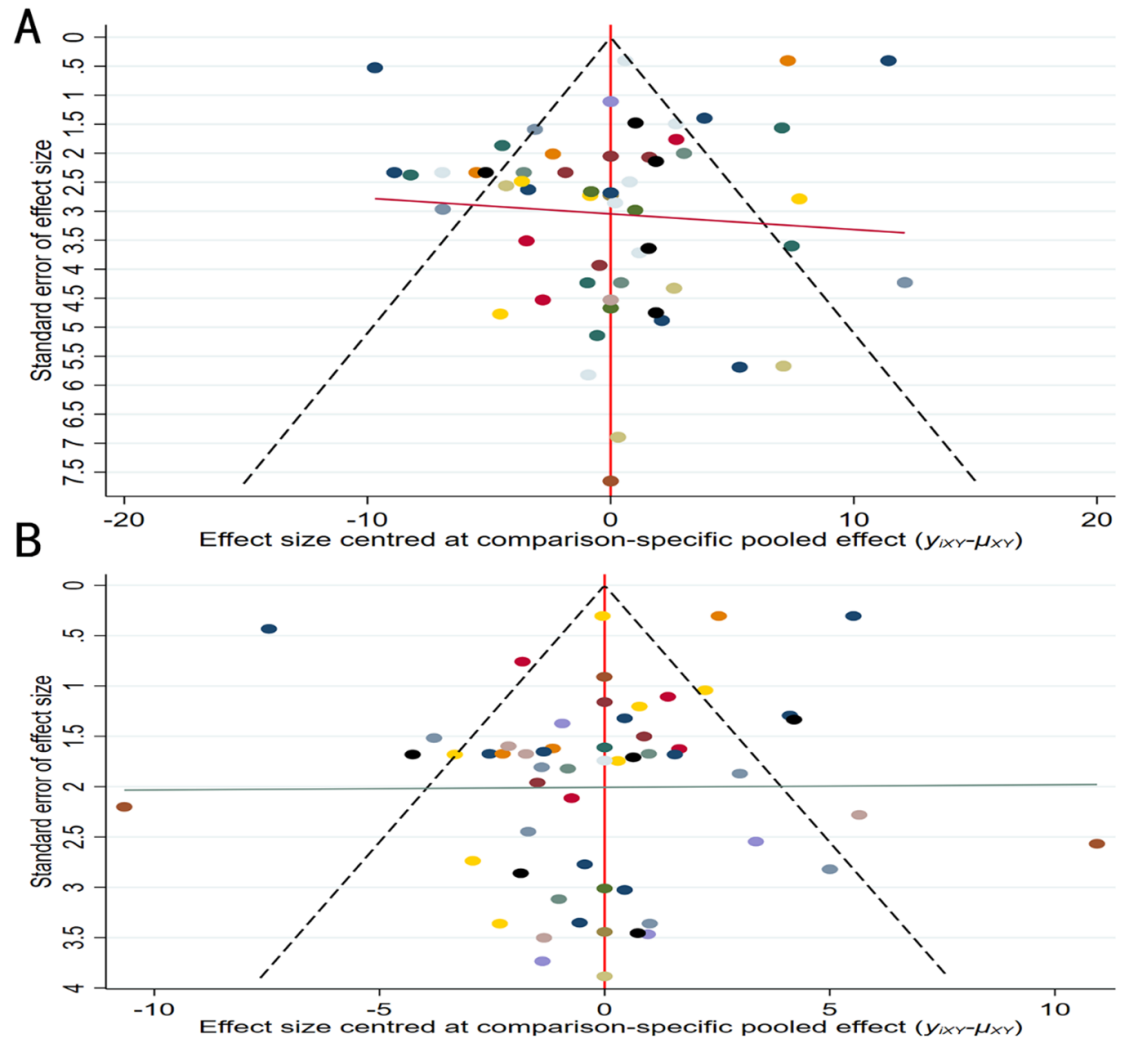
shows a publication bias funnel plot, where **(A)** represents SBP and **(B)** represents DBP.

Funnel plots were generated for each outcome to assess small-study effects and the risk of publication bias. The scatter of studies appeared broadly symmetrical, with no clear systematic asymmetry or extreme outliers, suggesting no obvious evidence of substantial publication bias (**Figure 8**).

### 3.7 Certainty of evidence (GRADE)

The certainty of evidence for the network estimates was assessed within the GRADE framework using CINeMA (**Supplementary Tables 11**, **12**). Across the two outcome networks, certainty ratings for 90 comparisons were distributed as follows: 17 high-certainty, 4 moderate-certainty, 20 low-certainty, and 49 very-low-certainty comparisons. Among the principal findings, the certainty of evidence was rated as high for the reduction in systolic blood pressure with combined training (CT) versus standard care (SOC), and for the reduction in diastolic blood pressure with both high-intensity continuous training (HICT) and CT versus SOC. A higher SUCRA ranking does not necessarily indicate greater certainty of evidence. Intervention rankings should therefore be interpreted in conjunction with the effect estimates, 95% confidence intervals, and the GRADE/CINeMA assessments. Comparisons with higher certainty were typically supported by multiple direct head-to-head trials, with confidence intervals that did not cross the line of no effect or the minimal important difference (MID) threshold. Low- or very-low-certainty comparisons were generally attributable to sparse networks or predominant reliance on indirect evidence, often accompanied by wide confidence intervals or potential small-study effects.

## 4 Discussion

### 4.1 Main findings

Although several exercise modalities are used to lower blood pressure in patients with hypertension, evidence from direct and indirect comparisons remains limited. We therefore conducted a network meta-analysis to synthesize both types of evidence and compare the relative effects of HICT, HIIT, LICT, MICT, MIIT, FFT, IFT, RT, and CT, with SOC as the comparator. The review included 30 randomized controlled trials involving 2,137 patients with hypertension. High-certainty evidence showed that CT significantly reduced blood pressure compared with SOC. For diastolic blood pressure, HICT appeared to provide a slightly larger reduction than CT, also supported by high-certainty evidence (47).High-certainty evidence also supported the blood pressure-lowering effects of isometric handgrip training (IFT) and moderate-intensity continuous training (MICT) (48).These interventions may also be considered useful blood pressure-lowering options, although they ranked somewhat lower. Taken together, the findings suggest that multicomponent combined training programmes centered on moderate- to high-intensity exercise may represent a promising strategy for improving blood pressure control and reducing cardiovascular risk in patients with hypertension (49).

### 4.2 Interpretation of the findings

The effect of combined training (CT) in improving blood pressure may be associated with the following mechanisms:

1. Improve vascular endothelial function and enhance vasodilation.
  The aerobic component of combined training (CT) increases blood flow–induced shear stress, which stimulates endothelial release of nitric oxide (NO). NO promotes relaxation of vascular smooth muscle, thereby reducing peripheral vascular resistance and ultimately lowering blood pressure. Previous studies have also suggested that combined exercise may reduce blood pressure by increasing NO bioavailability, attenuating inflammatory responses, and improving vascular tone (50).
2. Reducing sympathetic drive and enhancing vagal tone.
  Patients with hypertension often exhibit heightened sympathetic activity and autonomic imbalance. Combined training (CT) may improve cardiovascular autonomic regulation by reducing sympathetic drive and enhancing parasympathetic, particularly vagal, activity, thereby lowering resting heart rate, vasoconstrictor tone, and blood pressure burden. Previous studies have shown that combined training can improve blood pressure, arterial stiffness, vagal activity, cardiorespiratory fitness, and lower-limb muscle strength (51).
3. Improving arterial stiffness and reducing peripheral resistance.
  Long-standing hypertension contributes to vascular remodeling and increased arterial stiffness. Through its aerobic component, combined training (CT) may improve vascular compliance, while resistance training may enhance skeletal muscle pump function and metabolic capacity. Together, these adaptations can reduce arterial stiffness and peripheral vascular resistance, thereby lowering systolic blood pressure load (52).
4. Suppressing the RAAS and vasoconstriction-related pathways.
  Exercise training may attenuate activity of the renin–angiotensin–aldosterone system (RAAS) and the sympathetic nervous system. Reduced RAAS activity may blunt angiotensin II–mediated vasoconstriction, sodium and water retention, and vascular remodeling, thereby supporting long-term blood pressure control (53).
5. Improving body composition, insulin resistance, and inflammation.
  Resistance training can increase skeletal muscle mass and resting metabolic rate, whereas aerobic training can reduce fat mass. When combined, these modalities may improve adiposity, insulin resistance, dyslipidemia, and chronic low-grade inflammation, all of which are implicated in the development and progression of hypertension. The blood pressure-lowering effect of combined training (CT) may therefore partly reflect a broader reduction in cardiometabolic risk. Some studies have suggested that combined training provides more comprehensive benefits than aerobic or resistance training alone, including improvements in blood pressure, cardiorespiratory fitness, and body composition (54).

### 4.3 Implications for clinical practice and future research

Our findings reinforce the role of exercise training as a key strategy for blood pressure control in patients with hypertension. As a feasible first-line non-pharmacological option, exercise offers a practical, evidence-informed means of improving cardiovascular health in this population. By bringing together comparative evidence across exercise modalities, this review provides a basis for more targeted and clinically applicable exercise recommendations.

Exercise training is inexpensive, relatively safe, and scalable. Its effects, however, are likely to vary with baseline fitness and exercise tolerance, underscoring the need for individualized prescription. Resistance training may enhance muscular strength and physical function through mechanical loading and stretch-mediated adaptations. Compared with single-modality programmes, combined aerobic and resistance training and functional fitness training may deliver broader health benefits. For individuals with low baseline fitness, low- to moderate-intensity training may be the most appropriate starting point, with intensity progressed gradually as tolerance improves.

### 4.4 Advantages and limitations

This review has several strengths. It used a network meta-analysis framework to synthesize direct and indirect evidence and compare multiple exercise interventions for blood pressure control in patients with hypertension. All included studies were randomized controlled trials, and both systolic and diastolic blood pressure were examined. We also classified exercise intensity and intervention type in detail, assessed risk of bias with RoB 2, and graded the certainty of evidence using CINeMA, which strengthened the transparency and interpretability of the findings. Additional analyses, including inconsistency testing, sensitivity analyses, meta-regression, and assessment of publication bias, supported the robustness of the results.

Several limitations should be noted. Although the evidence base was substantial, direct evidence remained sparse for some interventions and outcomes, and several network estimates were driven largely by indirect comparisons; therefore, the rankings should be interpreted with caution. Clinical heterogeneity may have been introduced by differences in age, sex, baseline blood pressure, exercise frequency, intervention duration, training intensity, and adherence. Blinding is inherently difficult in exercise trials, and bias related to randomization, medication adherence, or outcome reporting cannot be excluded. When change-score SDs were unavailable, they were derived from baseline and endpoint data, which may have added statistical uncertainty. Finally, this review focused mainly on blood pressure outcomes, with limited evidence on quality of life, adverse events, and long-term medication adherence. Most included studies enrolled middle-aged and older adults with hypertension, and the interventions were generally delivered under relatively controlled trial conditions. The applicability of these findings to younger individuals, patients with severe or poorly controlled hypertension, those with multimorbidity, and populations treated in routine clinical or community settings therefore requires further evaluation. Larger, rigorously designed randomized trials with longer follow-up are needed to clarify the long-term effects of different exercise modalities on blood pressure control in patients with hypertension.

## 5 Conclusion

This systematic review and network meta-analysis included 30 randomized controlled trials involving 2,137 patients with hypertension and compared multiple exercise modalities for reducing systolic and diastolic blood pressure. Overall, exercise training appears to be an effective non-pharmacological strategy for improving blood pressure control in this population. Combined training (CT) showed the most favorable profile for systolic blood pressure reduction, whereas high-intensity continuous training (HICT) ranked highest for diastolic blood pressure reduction. Isometric handgrip training (IFT) and moderate-intensity continuous training (MICT) also showed favorable blood pressure-lowering effects. Taking both efficacy rankings and clinical feasibility into account, CT may represent a preferred exercise strategy for blood pressure management in patients with hypertension. Nevertheless, evidence remains limited for some comparisons, and larger, well-designed randomized controlled trials with longer follow-up are needed to confirm the long-term efficacy and safety of these interventions.

## Data Availability

The original contributions presented in the study are included in the article/**Supplementary Material**. Further inquiries can be directed to the corresponding authors.
