# Supplementary material for: Non-pharmacological interventions for blood pressure management in hypertensive patients: selection of exercise regimens—a systematic review and network meta-analysis
Source: Front Endocrinol (Lausanne). 2026 Aug 3;17:1926728. doi: 10.3389/fendo.2026.1926728 (PMC13477928; doi:10.3389/fendo.2026.1926728)
Supplement: Supplementary file 1 [file Table1.docx]

Table S1	PRISMA NMA Checklist of Items to Include When Reporting a Systematic Review Involving a Network Meta-analysis

| Section/Topic | Checklist Item |
| --- | --- |
| TITLE |  |
| Title | Non-pharmacological interventions for blood pressure management in hypertensive patients: Selection of exercise regimens—a systematic review and network meta-analysis |
|  |  |
| ABSTRACT |  |
| Structured summary | Provide a structured summary including, as applicable:  **Background:** main objectives  **Methods:** data sources; study eligibility criteria, participants, and interventions; study appraisal; and *synthesis methods, such as network meta-analysis.*  **Results:** number of studies and participants identified; summary estimates with corresponding confidence/credible intervals; treatment rankings may also be discussed. Authors may choose to summarize pairwise comparisons against a chosen treatment included in their analyses for brevity.  **Discussion/Conclusions:** limitations; conclusions and implications of findings.  **Other:** systematic review registration number with registry name. |
|  |  |
| INTRODUCTION |  |
| Rationale | Describe the rationale for the review in the context of what is already known*, including mention of why a network meta-analysis has been conducted.* |
| Objectives | Provide an explicit statement of questions being addressed, with reference to participants, interventions, comparisons, outcomes, and study design (PICOS). |
|  |  |
| METHODS |  |
| Protocol and registration | Indicate whether a review protocol exists and if and where it can be accessed (e.g., Web address); and, if available, provide registration information, including registration number. |
| Eligibility criteria | Specify study characteristics (e.g., PICOS, length of follow-up) and report characteristics (e.g., years considered, language, publication status) used as criteria for eligibility, giving rationale. *Clearly describe eligible treatments included in the treatment network, and note whether any have been clustered or merged into the same node (with justification).* |
| Information sources | Describe all information sources (e.g., databases with dates of coverage, contact with study authors to identify additional studies) in the search and date last searched. |
| Search | Present full electronic search strategy for at least one database, including any limits used, such that it could be repeated. |
| Study selection | State the process for selecting studies (i.e., screening, eligibility, included in systematic review, and, if applicable, included in the meta-analysis). |
| Data collection process | Describe method of data extraction from reports (e.g., piloted forms, independently, in duplicate) and any processes for obtaining and confirming data from investigators. |
| Data items | List and define all variables for which data were sought (e.g., PICOS, funding sources) and any assumptions and simplifications made. |
| Geometry of the network | Describe methods used to explore the geometry of the treatment network under study and potential biases related to it. This should include how the evidence base has been graphically summarized for presentation, and what characteristics were compiled and used to describe the evidence base to readers. |
| Risk of bias within individual studies | Describe methods used for assessing risk of bias of individual studies (including specification of whether this was done at the study or outcome level), and how this information is to be used in any data synthesis. |
| Summary measures | State the principal summary measures (e.g., risk ratio, difference in means). Also describe the use of additional summary measures assessed, such as treatment rankings and surface under the cumulative ranking curve (SUCRA) values, as well as modified approaches used to present summary findings from meta-analyses. |
| Planned methods of analysis | Describe the methods of handling data and combining results of studies for each network meta-analysis. This should include, but not be limited to:   - Handling of multi-arm trials; - Selection of variance structure; - Selection of prior distributions in Bayesian analyses; and - Assessment of model fit. |
| Assessment of Inconsistency | Describe the statistical methods used to evaluate the agreement of direct and indirect evidence in the treatment network(s) studied. Describe efforts taken to address its presence when found. |
| Risk of bias across studies | Specify any assessment of risk of bias that may affect the cumulative evidence (e.g., publication bias, selective reporting within studies). |
| Additional analyses | Describe methods of additional analyses if done, indicating which were pre-specified. This may include, but not be limited to, the following:   - Sensitivity or subgroup analyses; - Meta-regression analyses; - Alternative formulations of the treatment network; and - Use of alternative prior distributions for Bayesian analyses (if applicable). |
| RESULTS† |  |
| Study selection | Give numbers of studies screened, assessed for eligibility, and included in the review, with reasons for exclusions at each stage, ideally with a flow diagram. |
| Presentation of network structure | Provide a network graph of the included studies to enable visualization of the geometry of the treatment network. |
| Summary of network geometry | Provide a brief overview of characteristics of the treatment network. This may include commentary on the abundance of trials and randomized patients for the different interventions and pairwise comparisons in the network, gaps of evidence in the treatment network, and potential biases reflected by the network structure. |
| Study characteristics | For each study, present characteristics for which data were extracted (e.g., study size, PICOS, follow-up period) and provide the citations. |
| Risk of bias within studies | Present data on risk of bias of each study and, if available, any outcome level assessment. |
| Results of individual studies | For all outcomes considered (benefits or harms), present, for each study: 1) simple summary data for each intervention group, and 2) effect estimates and confidence intervals. *Modified approaches may be needed to deal with information from larger networks.* |
| Synthesis of results | Present results of each meta-analysis done, including confidence/credible intervals. In larger networks, authors may focus on comparisons versus a particular comparator (e.g. placebo or standard care), with full findings presented in an appendix. League tables and forest plots may be considered to summarize pairwise comparisons. If additional summary measures were explored (such as treatment rankings), these should also be presented. |
| Exploration for inconsistency | Describe results from investigations of inconsistency. This may include such information as measures of model fit to compare consistency and inconsistency models, *P* values from statistical tests, or summary of inconsistency estimates from different parts of the treatment network. |
| Risk of bias across studies | Present results of any assessment of risk of bias across studies for the evidence base being studied. |
| Results of additional analyses | Give results of additional analyses, if done (e.g., sensitivity or subgroup analyses, meta-regression analyses*, alternative network geometries studied, alternative choice of prior distributions for Bayesian analyses,* and so forth). |
|  |  |
| DISCUSSION |  |
| Summary of evidence | Summarize the main findings, including the strength of evidence for each main outcome; consider their relevance to key groups (e.g., healthcare providers, users, and policy-makers). |
| Limitations | Discuss limitations at study and outcome level (e.g., risk of bias), and at review level (e.g., incomplete retrieval of identified research, reporting bias). *Comment on the validity of the assumptions, such as transitivity and consistency. Comment on any concerns regarding network geometry (e.g., avoidance of certain comparisons).* |
| Conclusions | Provide a general interpretation of the results in the context of other evidence, and implications for future research. |
|  |  |
| FUNDING |  |
| Funding | Describe sources of funding for the systematic review and other support (e.g., supply of data); role of funders for the systematic review. This should also include information regarding whether funding has been received from manufacturers of treatments in the network and/or whether some of the authors are content experts with professional conflicts of interest that could affect use of treatments in the network. |

PICOS = population, intervention, comparators, outcomes, study design.

| **Table S2.Literature Search Strategy** | |
| --- | --- |
| **Pubmed** | #1 "Hypertension"[Mesh] Most Recent "Hypertension"[MeSH Terms] 338,757 1:24:43 2026/5/24  #2 High Blood Pressures Most Recent "hypertension"[MeSH Terms] OR "hypertension"[All Fields] OR ("high"[All Fields] AND "blood"[All Fields] AND "pressures"[All Fields]) OR "high blood pressures"[All Fields] 691,321 1:25:31 2026/5/24  #3 ("Hypertension"[Mesh]) OR (High Blood Pressures) "Hypertension"[MeSH Terms] OR ("Hypertension"[MeSH Terms] OR "Hypertension"[All Fields] OR ("high"[All Fields] AND "blood"[All Fields] AND "pressures"[All Fields]) OR "high blood pressures"[All Fields]) 691,321 1:26:05 2026/5/24  #4 Blood Pressure[Title/Abstract] "blood pressure"[Title/Abstract] 387,131 1:27:08 2026/5/24  #5 (("Hypertension"[Mesh]) OR (High Blood Pressures)) OR (Blood Pressure[Title/Abstract]) "Hypertension"[MeSH Terms] OR ("Hypertension"[MeSH Terms] OR "Hypertension"[All Fields] OR ("high"[All Fields] AND "Blood"[All Fields] AND "pressures"[All Fields]) OR "high blood pressures"[All Fields]) OR "blood pressure"[Title/Abstract] 913,338 1:27:46 2026/5/24  #6 "Exercise"[Mesh]OR Physical Activity Most Recent "Exercise"[MeSH Terms] OR ("Exercise"[MeSH Terms] OR "Exercise"[All Fields] OR ("physical"[All Fields] AND "activity"[All Fields]) OR "physical activity"[All Fields]) 835,113 1:29:37 2026/5/24  #7 "Therapeutics"[Mesh] Most Recent "Therapeutics"[MeSH Terms] 5,675,107 1:35:39 2026/5/24  #8 (SBP[Title/Abstract]) OR (DBP[Title/Abstract]) "SBP"[Title/Abstract] OR "DBP"[Title/Abstract] 43,780 1:36:17 2026/5/24  #9 ((((("Hypertension"[Mesh]) OR (High Blood Pressures)) OR (Blood Pressure[Title/Abstract])) AND ("Exercise"[Mesh]OR Physical Activity)) AND ("Therapeutics"[Mesh])) AND ((SBP[Title/Abstract]) OR (DBP[Title/Abstract])) ("Hypertension"[MeSH Terms] OR ("Hypertension"[MeSH Terms] OR "Hypertension"[All Fields] OR ("high"[All Fields] AND "Blood"[All Fields] AND "pressures"[All Fields]) OR "high blood pressures"[All Fields]) OR "blood pressure"[Title/Abstract]) AND ("Exercise"[MeSH Terms] OR ("Exercise"[MeSH Terms] OR "Exercise"[All Fields] OR ("physical"[All Fields] AND "activity"[All Fields]) OR "physical activity"[All Fields])) AND "Therapeutics"[MeSH Terms] AND ("SBP"[Title/Abstract] OR "DBP"[Title/Abstract]) 731 1:36:51 2026/5/24  #10 ((((("Hypertension"[Mesh]) OR (High Blood Pressures)) OR (Blood Pressure[Title/Abstract])) AND ("Exercise"[Mesh]OR Physical Activity)) AND ("Therapeutics"[Mesh])) AND ((SBP[Title/Abstract]) OR (DBP[Title/Abstract])) Randomized Controlled Trial (("Hypertension"[MeSH Terms] OR ("Hypertension"[MeSH Terms] OR "Hypertension"[All Fields] OR ("high"[All Fields] AND "Blood"[All Fields] AND "pressures"[All Fields]) OR "high blood pressures"[All Fields]) OR "blood pressure"[Title/Abstract]) AND ("Exercise"[MeSH Terms] OR ("Exercise"[MeSH Terms] OR "Exercise"[All Fields] OR ("physical"[All Fields] AND "activity"[All Fields]) OR "physical activity"[All Fields])) AND "Therapeutics"[MeSH Terms] AND ("SBP"[Title/Abstract] OR "DBP"[Title/Abstract])) AND (randomizedcontrolledtrial[Filter]) 277 1:37:05 2026/5/24 |
| **Web of Science** | 1："ALL=(Hypertension OR ""Blood Pressure"" OR ""high blood pressure"") " Web of Science Core Collection 760800 Sun May 24 2026 14:17:47 GMT+0800 (中国标准时间)  "TS=(  (""exercise training"")  OR  (""exercise intervention"")  OR  (""physical activity intervention"")  OR  (""aerobic training"")  OR  (""resistance training"")  OR  (""strength training"")  OR  (""endurance training"")  OR  (""combined training"")  OR  (""multicomponent training"")  OR  (""exercise therap*"")) "  2: "ALL=(""physical activity"" OR Exercise) " Web of Science Core Collection 902994 Sun May 24 2026 14:19:10 GMT+0800 (中国标准时间)  3："TS=(Therapeutics) " Web of Science Core Collection 151678 Sun May 24 2026 14:20:01 GMT+0800 (中国标准时间) "  4："TS=(SBP OR DBP) " Web of Science Core Collection 43415 Sun May 24 2026 14:20:26 GMT+0800 (中国标准时间)  5"(((#1) AND TS=(""physical activity"" OR Exercise)) AND TS=(Therapeutics)) AND TS=(SBP OR DBP) " Web of Science Core Collection 5 Sun May 24 2026 14:24:30 GMT+0800 (中国标准时间)   6 "#1 AND #2 AND #3 " Web of Science Core Collection 142 Sun May 24 2026 14:25:40 GMT+0800 (中国标准时间) 7 "TS=(   random* OR randomi* OR randomly  OR placebo* OR sham  OR trial OR ""clinical trial"" OR ""controlled clinical trial""  OR ""double blind"" OR ""single blind"" OR ""triple blind""  OR crossover OR ""cross over"" OR ""parallel group""  OR cluster random*  OR RCT) " Web of Science Core Collection 4080133 Sun May 24 2026 14:27:53 GMT+0800 (中国标准时间)  8. "#6 AND #7 " Web of Science Core Collection 62 Sun May 24 2026 14:28:57 GMT+0800 (中国标准时间) |
| **Cochrane** | #1 MeSH descriptor: [Hypertension] explode all trees 26285  #2 Blood Pressure OR High Blood Pressures 150335  #3 #1 OR #2 157620  #4 MeSH descriptor: [Exercise] explode all trees 43179  #5 MeSH descriptor: [Exercise Therapy] explode all trees 24903  #6 SBP OR DBP 14981  #7 #3 AND #4 AND #5 AND #6 166 |

Table of P-values for each outcome metric

Table S3: Global inconsistency table of SBP and DBP results in hypertensive patients

| **End Point Indicator Name** | **P-value for global inconsistency analysis** |
| --- | --- |
| SBP | 0.9556 |
| DBP | 0.9973 |

Node splitting analysis table

S4. Node splitting analysis of the outcome endpoint SBP

| **Side** | **Coef.** | **Std. Err.** | **Coef.** | **Std. Err.** | **Coef.** | **Std. Err.** | **P>z** | **tau** |
| --- | --- | --- | --- | --- | --- | --- | --- | --- |
| CT VS HICT | 5.131809 | 5.579063 | 3.006521 | 7.268854 | 2.125288 | 7.268854 | 0.77 | 5.188347 |
| CT VS IFT | 5.981067 | 6.961855 | 3.697382 | 7.711488 | 2.283685 | 7.711488 | 0.767 | 5.169815 |
| CT VS MICT | 5.772411 | 3.064467 | 0.2924673 | 4.911643 | 5.479944 | 4.911643 | 0.265 | 5.008433 |
| CT VS RT | 2.522795 | 3.422096 | 3.976509 | 5.754246 | -1.453714 | 5.754246 | 0.801 | 5.194406 |
| CT VS SOC | 8.691023 | 2.174789 | 14.66039 | 5.888322 | -5.969363 | 5.888322 | 0.311 | 5.101726 |
| FFT VS MICT | 2.4 | 5.31722 | -0.2786638 | 6.727548 | 2.678664 | 6.727548 | 0.691 | 5.200269 |
| FFT VS SOC | 5.884069 | 3.546183 | 8.56275 | 6.727529 | -2.67868 | 6.727529 | 0.691 | 5.200256 |
| HICT VS MICT | -0.9562874 | 9.174216 | -0.1364642 | 9.942915 | -0.8198232 | 9.942915 | 0.934 | 5.151357 |
| HICT VS SOC | 5.393072 | 3.479085 | 7.347106 | 9.469757 | -1.954034 | 9.469757 | 0.837 | 5.180426 |
| HIIT VS SOC | 6.536139 | 3.408546 | 19.20209 | 2239.887 | -12.66595 | 2239.887 | 0.995 | 5.106455 |
| IFT VS LICT | -6.528228 | 5.823438 | -1.059013 | 7.291112 | -5.469216 | 7.291112 | 0.453 | 5.14592 |
| IFT VS RT | -2.979745 | 6.864452 | -0.5457813 | 7.778191 | -2.433964 | 7.778191 | 0.754 | 5.171204 |
| IFT VS SOC | 5.51853 | 2.469729 | 4.119026 | 8.426233 | 1.399504 | 8.426233 | 0.868 | 5.184993 |
| LICT VS SOC | 7.42856 | 2.963628 | 23.42131 | 11.39102 | -15.99275 | 11.39102 | 0.16 | 5.013792 |
| MICT VS MIIT | 1.812083 | 5.825416 | -2.222715 | 8.175019 | 4.034797 | 8.175019 | 0.622 | 5.171507 |
| MICT VS RT | 0.6557106 | 3.382174 | -2.757139 | 5.616963 | 3.41285 | 5.616963 | 0.543 | 5.155112 |
| MICT VS SOC | 6.621326 | 2.19854 | 3.107282 | 4.79826 | 3.514044 | 4.79826 | 0.464 | 5.111863 |
| MIIT VS SOC | 6.771424 | 4.170145 | 1.62116 | 11.61045 | 5.150265 | 11.61045 | 0.657 | 5.17816 |
| RT VS SOC | 6.286664 | 2.654035 | 7.403879 | 6.318227 | -1.117215 | 6.318227 | 0.86 | 5.196768 |

Table S5. Node splitting analysis of the outcome endpoint DBP

| **Side** | **Coef.** | **Std. Err.** | **Coef.** | **Std. Err.** | **Coef.** | **Std. Err.** | **P>z** | **tau** |
| --- | --- | --- | --- | --- | --- | --- | --- | --- |
| CT VS HICT | -0.1053098 | 3.711923 | -2.635883 | 2.963662 | 2.530574 | 4.74724 | 0.594 | 3.525971 |
| CT VS IFT | 2.008359 | 4.628894 | 2.798884 | 2.306922 | -0.7905252 | 5.159773 | 0.878 | 3.527847 |
| CT VS MICT | 4.149222 | 2.093797 | -0.1361599 | 2.620916 | 4.285382 | 3.358466 | 0.202 | 3.380737 |
| CT VS RT | 2.831583 | 2.401485 | 2.410102 | 3.173131 | 0.4214813 | 3.969499 | 0.915 | 3.541726 |
| CT VS SOC | 5.592983 | 1.480959 | 7.449842 | 3.829178 | -1.856859 | 4.107776 | 0.651 | 3.523063 |
| FFT VS SOC | 3.098947 | 1.881982 | 11.46512 | 2240.065 | -8.366169 | 2240.066 | 0.997 | 3.476954 |
| HICT VSG | 6.00677 | 5.235435 | 3.632626 | 2.682318 | 2.374144 | 5.877249 | 0.686 | 3.51208 |
| HICT VS SOC | 7.494084 | 2.232116 | 7.441636 | 5.775972 | 0.0524474 | 6.160378 | 0.993 | 3.540116 |
| HIIT VS SOC | 2.828555 | 2.294847 | 11.63485 | 1419.035 | -8.806298 | 1419.037 | 0.995 | 3.476955 |
| IFT VS LICT | -1.336786 | 3.879539 | 0.8324935 | 3.074205 | -2.169279 | 4.947343 | 0.661 | 3.529637 |
| IFT VS RT | 0.020925 | 4.926087 | 0.0390028 | 2.565815 | -0.0180777 | 5.555217 | 0.997 | 3.5248 |
| IFT VS SOC | 3.108983 | 1.717824 | 4.04115 | 5.464529 | -0.9321666 | 5.729359 | 0.871 | 3.530572 |
| LICT VS SOC | 2.68311 | 2.068045 | 10.15977 | 7.556265 | -7.476665 | 7.823223 | 0.339 | 3.481886 |
| MICT VS MIIT | 3.280328 | 3.921895 | -0.5140274 | 3.814705 | 3.794356 | 5.443357 | 0.486 | 3.515506 |
| MICT VS RT | 0.4142714 | 2.355905 | -0.194448 | 3.221698 | 0.6087194 | 3.984202 | 0.879 | 3.539922 |
| MICT VS SOC | 3.863698 | 1.461834 | -0.1771437 | 3.86557 | 4.040842 | 4.125805 | 0.327 | 3.432473 |
| MIIT VS SOC | 2.575673 | 2.77877 | -1.839754 | 7.403594 | 4.415427 | 7.908472 | 0.577 | 3.526444 |
| RT VS SOC | 3.291644 | 1.85935 | 2.518075 | 3.976857 | 0.7735687 | 4.357105 | 0.859 | 3.537841 |

**SUCRA value**

**Table S6: SUCRA values for the outcome parameter SBP**

| **Treatm~t** | **SUCRA** | **PrBest** | **MeanRank** |
| --- | --- | --- | --- |
| CT | 83.8 | 32.5 | 2.5 |
| LICT | 72.1 | 24.7 | 3.5 |
| FFT | 54.1 | 8.6 | 5.1 |
| HIIT | 53.8 | 11.4 | 5.2 |
| RT | 52.5 | 3.7 | 5.3 |
| MIIT | 49.7 | 11.5 | 5.5 |
| MICT | 46 | 0.9 | 5.9 |
| HICT | 44.7 | 5.5 | 6 |
| IFT | 41.6 | 1.3 | 6.3 |
| SOC | 1.7 | 0 | 9.8 |

**Table S7: SUCRA values for the outcome parameter DBP**

| **Treatm~t** | **SUCRA** | **PrBest** | **MeanRank** |
| --- | --- | --- | --- |
| HICT | 93.4 | 68.8 | 1.6 |
| CT | 83.3 | 17.2 | 2.5 |
| MICT | 49.9 | 0.5 | 5.5 |
| LICT | 47.9 | 2.8 | 5.7 |
| IFT | 47.3 | 1.3 | 5.7 |
| RT | 47.1 | 1.2 | 5.8 |
| FFT | 47 | 2.4 | 5.8 |
| HIIT | 44.4 | 3.6 | 6 |
| MIIT | 34.4 | 2.3 | 6.9 |
| SOC | 5.5 | 0 | 9.5 |

**Meta-analysis**

**Table S8 presents a meta-regression analysis using the average age of patients as the moderating variable.**

| **Intervention** | **Covariate** | **Coefficient** | **Standard** Error | **Z-statistic** | **P>z** | **[95% conf.** | **interval]** |
| --- | --- | --- | --- | --- | --- | --- | --- |
| CT vs FFT | age | -0.0467325 | 0.4372008 | -0.11 | 0.915 | -0.9036303 | 0.8101652 |
| CT vs FFT | _cons | 5.938809 | 24.01859 | 0.25 | 0.805 | -41.13676 | 53.01438 |
| CT vs HICT | age | -0.397593 | 0.5160764 | -0.77 | 0.441 | -1.409084 | 0.6138981 |
| CT vs HICT | _cons | 25.08083 | 26.76611 | 0.94 | 0.349 | -27.37979 | 77.54144 |
| CT vs HIIT | age | -0.7744016 | 2.206132 | -0.35 | 0.726 | -5.09834 | 3.549537 |
| CT vs HIIT | _cons | 48.05775 | 132.1232 | 0.36 | 0.716 | -210.899 | 307.0145 |
| CT vs IFT | age | 0.0036313 | 0.3775397 | 0.01 | 0.992 | -0.7363329 | 0.7435956 |
| CT vs IFT | _cons | 3.095467 | 21.62029 | 0.14 | 0.886 | -39.27952 | 45.47045 |
| CT vs LICT | age | 0.2109334 | 0.4789032 | 0.44 | 0.66 | -0.7276997 | 1.149567 |
| CT vs LICT | _cons | -14.43474 | 29.55325 | -0.49 | 0.625 | -72.35804 | 43.48856 |
| CT VS MICT | age | -0.306465 | 0.3367703 | -0.91 | 0.363 | -0.9665226 | 0.3535927 |
| CT vs MICT | _cons | 20.3432 | 18.89396 | 1.08 | 0.282 | -16.68829 | 57.37469 |
| CT vs MIIT | age | 0.7555663 | 3.389598 | 0.22 | 0.824 | -5.887924 | 7.399057 |
| CT vs MIIT | _cons | -50.73297 | 226.4594 | -0.22 | 0.823 | -494.5852 | 393.1192 |
| CT vs RT | age | -0.3219704 | 0.3944378 | -0.82 | 0.414 | -1.095054 | 0.4511134 |
| CT vs RT | _cons | 20.5542 | 21.61001 | 0.95 | 0.342 | -21.80063 | 62.90904 |
| CT vs SOC | age | -0.3190725 | 0.2510528 | -1.27 | 0.204 | -0.811127 | 0.1729819 |
| CT vs SOC | _cons | 27.35587 | 14.16991 | 1.93 | 0.054 | -0.4166456 | 55.12839 |

**Table S9 presents a meta-regression analysis using intervention time as the moderating variable.**

| **Intervention** | **Covariate** | **Coefficient** | **Standard** Error | **Z-statistic** | **P>z** | **[95% conf.** | **interval]** |
| --- | --- | --- | --- | --- | --- | --- | --- |
| CT vs FFT | time | -0.0801922 | 0.4894601 | -0.16 | 0.87 | -1.039516 | 0.8791321 |
| CT vs FFT | _cons | 4.375169 | 8.201261 | 0.53 | 0.594 | -11.69901 | 20.44934 |
| CT vs HICT | time | 0.2113386 | 0.5173239 | 0.41 | 0.683 | -0.8025977 | 1.225275 |
| CT vs HICT | _cons | -0.6566028 | 9.733942 | -0.07 | 0.946 | -19.73478 | 18.42157 |
| CT vs HIIT | time | -3.46944 | 2.328178 | -1.49 | 0.136 | -8.032585 | 1.093705 |
| CT vs HIIT | _cons | 29.74494 | 19.08232 | 1.56 | 0.119 | -7.655721 | 67.1456 |
| CT vs IFT | time | -1.019268 | 1.591481 | -0.64 | 0.522 | -4.138513 | 2.099976 |
| CT vs IFT | _cons | 14.48531 | 16.89959 | 0.86 | 0.391 | -18.63727 | 47.60789 |
| CT vs LICT | time | 0.0498149 | 0.5226742 | 0.1 | 0.924 | -0.9746076 | 1.074237 |
| CT vs LICT | _cons | -0.2344307 | 8.402664 | -0.03 | 0.978 | -16.70335 | 16.23449 |
| CT VS MICT | time | -0.0382485 | 0.4594904 | -0.08 | 0.934 | -0.9388332 | 0.8623361 |
| CT vs MICT | _cons | 4.107418 | 6.512944 | 0.63 | 0.528 | -8.657718 | 16.87255 |
| CT vs MIIT | time | 0.3217951 | 1.150245 | 0.28 | 0.78 | -1.932644 | 2.576235 |
| CT vs MIIT | _cons | -0.7752934 | 15.2568 | -0.05 | 0.959 | -30.67808 | 29.12749 |
| CT vs RT | time | -0.0274439 | 0.481585 | -0.06 | 0.955 | -0.9713331 | 0.9164454 |
| CT vs RT | _cons | 3.303719 | 6.647811 | 0.5 | 0.619 | -9.725751 | 16.33319 |
| CT vs SOC | time | -0.0202736 | 0.4361448 | -0.05 | 0.963 | -0.8751016 | 0.8345545 |
| CT vs SOC | _cons | 9.661267 | 5.972675 | 1.62 | 0.106 | -2.04496 | 21.36749 |

**Sensitivity Analysis**

**Table S10: Sensitivity Analysis**

| **dropped_id** | **comparison** | **eff** | **lci** | **uci** | **connected** |
| --- | --- | --- | --- | --- | --- |
| Alemayehu 2023 | SOC vs CT | -2.0343117 | -3.396142 | -0.5244987 | 1 |
| Alemayehu 2023 | SOC vs FFT | -0.52576172 | -2.255962 | 0.9267222 | 1 |
| Alemayehu 2023 | SOC vs HICT | -0.64656649 | -2.278148 | 0.1140615 | 1 |
| Alemayehu 2023 | SOC vs HIIT | -0.73900168 | -2.732455 | 1.022326 | 1 |
| Alemayehu 2023 | SOC vs FFT | -0.73848694 | -2.076451 | 0.5837553 | 1 |
| Alemayehu 2023 | SOC vs LICT | -0.7504088 | -2.413458 | 0.9478037 | 1 |
| Alemayehu 2023 | SOC vs MICT | -0.54057007 | -1.777215 | 0.5220999 | 1 |
| Alemayehu 2023 | SOC vs MIIT | -0.58424444 | -2.853713 | 1.4301 | 1 |
| Alemayehu 2023 | SOC vs RT | -1.0047353 | -2.564208 | 0.7252255 | 1 |
| Banks 2024 | SOC vs CT | -2.055865 | -3.281136 | -0.6780823 | 1 |
| Banks 2024 | SOC vs FFT | -0.56844394 | -2.233366 | 0.9024065 | 1 |
| Banks 2024 | SOC vs HICT | -0.67399114 | -2.246953 | 0.0640844 | 1 |
| Banks 2024 | SOC vs HIIT | -0.73891628 | -2.662616 | 0.9951137 | 1 |
| Banks 2024 | SOC vs FFT | -0.7343439 | -2.024954 | 0.5472593 | 1 |
| Banks 2024 | SOC vs LICT | -0.74989455 | -2.354131 | 0.9217987 | 1 |
| Banks 2024 | SOC vs MICT | -0.70973322 | -1.834243 | 0.3257023 | 1 |
| Banks 2024 | SOC vs MIIT | -0.63279707 | -2.818527 | 1.362446 | 1 |
| Banks 2024 | SOC vs RT | -0.93475412 | -2.41199 | 0.5045575 | 1 |
| Lopes 2021 | SOC vs CT | -2.0773153 | -3.303664 | -0.6678578 | 1 |
| Lopes 2021 | SOC vs FFT | -0.57411883 | -2.248747 | 0.8979701 | 1 |
| Lopes 2021 | SOC vs HICT | -0.68034094 | -2.260787 | 0.0734874 | 1 |
| Lopes 2021 | SOC vs HIIT | -0.73892625 | -2.670387 | 0.9901525 | 1 |
| Lopes 2021 | SOC vs FFT | -0.74190145 | -2.036732 | 0.5440138 | 1 |
| Lopes 2021 | SOC vs LICT | -0.7510382 | -2.361801 | 0.9176807 | 1 |
| Lopes 2021 | SOC vs MICT | -0.73230951 | -1.924758 | 0.452102 | 1 |
| Lopes 2021 | SOC vs MIIT | -0.63929216 | -2.836763 | 1.37949 | 1 |
| Lopes 2021 | SOC vs RT | -1.0150518 | -2.379333 | 0.401574 | 1 |
| Caminiti 2021 | SOC vs CT | -2.3977819 | -3.645601 | -1.087116 | 1 |
| Caminiti 2021 | SOC vs FFT | -0.5920317 | -2.164803 | 0.7484969 | 1 |
| Caminiti 2021 | SOC vs HICT | -0.73869518 | -2.230048 | -0.1443335 | 1 |
| Caminiti 2021 | SOC vs HIIT | -0.73877135 | -2.559252 | 0.8237225 | 1 |
| Caminiti 2021 | SOC vs FFT | -0.77095861 | -1.99173 | 0.3949367 | 1 |
| Caminiti 2021 | SOC vs LICT | -0.75570217 | -2.272865 | 0.7692862 | 1 |
| Caminiti 2021 | SOC vs MICT | -0.8026448 | -1.864963 | 0.1173987 | 1 |
| Caminiti 2021 | SOC vs MIIT | -0.65943508 | -2.725693 | 1.132279 | 1 |
| Caminiti 2021 | SOC vs RT | -1.1047077 | -2.392795 | 0.1567225 | 1 |
| Schroeder 2019 | SOC vs CT | -2.4320471 | -3.790055 | -0.7417961 | 1 |
| Schroeder 2019 | SOC vs FFT | -0.57515403 | -2.296391 | 0.9537426 | 1 |
| Schroeder 2019 | SOC vs HICT | -0.73338564 | -2.35683 | 0.0841469 | 1 |
| Schroeder 2019 | SOC vs HIIT | -0.73898979 | -2.722268 | 1.052602 | 1 |
| Schroeder 2019 | SOC vs FFT | -0.77218104 | -2.103322 | 0.5601531 | 1 |
| Schroeder 2019 | SOC vs LICT | -0.75558813 | -2.410062 | 0.9668994 | 1 |
| Schroeder 2019 | SOC vs MICT | -0.73671456 | -1.969513 | 0.4211104 | 1 |
| Schroeder 2019 | SOC vs MIIT | -0.64059428 | -2.898402 | 1.429987 | 1 |
| Schroeder 2019 | SOC vs RT | -1.0664381 | -2.621832 | 0.3903669 | 1 |
| Li 2024 | SOC vs CT | -2.0876472 | -3.310863 | -0.6893547 | 1 |
| Li 2024 | SOC vs FFT | -0.43454213 | -2.335664 | 1.030052 | 1 |
| Li 2024 | SOC vs HICT | -0.68842043 | -2.265568 | 0.0634361 | 1 |
| Li 2024 | SOC vs HIIT | -0.73892171 | -2.666835 | 0.9970374 | 1 |
| Li 2024 | SOC vs FFT | -0.74363779 | -2.036051 | 0.5453144 | 1 |
| Li 2024 | SOC vs LICT | -0.75131053 | -2.359081 | 0.923069 | 1 |
| Li 2024 | SOC vs MICT | -0.77845764 | -1.951113 | 0.3147221 | 1 |
| Li 2024 | SOC vs MIIT | -0.65255414 | -2.845136 | 1.362732 | 1 |
| Li 2024 | SOC vs RT | -1.0287354 | -2.389115 | 0.3774967 | 1 |
| Arija 2018 | SOC vs CT | -2.0756881 | -3.296391 | -0.6904032 | 1 |
| Arija 2018 | SOC vs FFT | -0.57262178 | -2.242024 | 0.9030953 | 1 |
| Arija 2018 | SOC vs HICT | -0.6792667 | -2.256108 | 0.06241 | 1 |
| Arija 2018 | SOC vs HIIT | -0.73892317 | -2.667973 | 0.9958842 | 1 |
| Arija 2018 | SOC vs FFT | -0.75207902 | -2.048262 | 0.5378301 | 1 |
| Arija 2018 | SOC vs LICT | -0.85749132 | -2.702633 | 1.022921 | 1 |
| Arija 2018 | SOC vs MICT | -0.72635148 | -1.84725 | 0.3137499 | 1 |
| Arija 2018 | SOC vs MIIT | -0.63757786 | -2.829207 | 1.361306 | 1 |
| Arija 2018 | SOC vs RT | -1.0139826 | -2.371103 | 0.3758879 | 1 |
| Fecchio 2023 | SOC vs CT | -2.3372152 | -3.738994 | -0.7787459 | 1 |
| Fecchio 2023 | SOC vs FFT | -0.5840419 | -2.347562 | 0.9568525 | 1 |
| Fecchio 2023 | SOC vs HICT | -0.7240845 | -2.389013 | 0.0732674 | 1 |
| Fecchio 2023 | SOC vs HIIT | -0.73904752 | -2.773237 | 1.056089 | 1 |
| Fecchio 2023 | SOC vs FFT | -0.68965757 | -2.224533 | 0.8239328 | 1 |
| Fecchio 2023 | SOC vs LICT | -0.7428607 | -2.443641 | 0.9920395 | 1 |
| Fecchio 2023 | SOC vs MICT | -0.77229715 | -1.966242 | 0.3147794 | 1 |
| Fecchio 2023 | SOC vs MIIT | -0.65084827 | -2.964463 | 1.418707 | 1 |
| Fecchio 2023 | SOC vs RT | -1.0409586 | -2.654365 | 0.5294296 | 1 |
| Sakairi 2025 | SOC vs CT | -2.0744867 | -3.2992 | -0.7125321 | 1 |
| Sakairi 2025 | SOC vs FFT | -0.57236737 | -2.243007 | 0.8931092 | 1 |
| Sakairi 2025 | SOC vs HICT | -0.67110631 | -2.460458 | 0.0185354 | 1 |
| Sakairi 2025 | SOC vs HIIT | -0.73892478 | -2.669238 | 0.9847177 | 1 |
| Sakairi 2025 | SOC vs FFT | -0.74151048 | -2.035497 | 0.5349839 | 1 |
| Sakairi 2025 | SOC vs LICT | -0.75098037 | -2.360774 | 0.9122226 | 1 |
| Sakairi 2025 | SOC vs MICT | -0.72534881 | -1.849593 | 0.2893018 | 1 |
| Sakairi 2025 | SOC vs MIIT | -0.6372906 | -2.830492 | 1.342575 | 1 |
| Sakairi 2025 | SOC vs RT | -1.0126981 | -2.371237 | 0.3602021 | 1 |
| Twerenbold 2023 | SOC vs CT | -2.0689652 | -3.281485 | -0.7115904 | 1 |
| Twerenbold 2023 | SOC vs FFT | -0.57240954 | -2.228104 | 0.8242357 | 1 |
| Twerenbold 2023 | SOC vs HICT | -0.67820212 | -2.24262 | -0.0032519 | 1 |
| Twerenbold 2023 | SOC vs HIIT | -1.1116941 | -3.458978 | 0.3965005 | 1 |
| Twerenbold 2023 | SOC vs FFT | -0.7408679 | -2.023615 | 0.4877436 | 1 |
| Twerenbold 2023 | SOC vs LICT | -0.75091216 | -2.346669 | 0.8471279 | 1 |
| Twerenbold 2023 | SOC vs MICT | -0.72541748 | -1.837358 | 0.2635911 | 1 |
| Twerenbold 2023 | SOC vs MIIT | -0.63729778 | -2.811146 | 1.259339 | 1 |
| Twerenbold 2023 | SOC vs RT | -1.0114419 | -2.35787 | 0.3232387 | 1 |
| Dimeo 2012 | SOC vs CT | -2.0736732 | -3.295913 | -0.6846931 | 1 |
| Dimeo 2012 | SOC vs FFT | -0.5706964 | -2.242128 | 0.9015363 | 1 |
| Dimeo 2012 | SOC vs HICT | -0.6778793 | -2.256414 | 0.0649588 | 1 |
| Dimeo 2012 | SOC vs HIIT | -0.73892551 | -2.669807 | 0.9941405 | 1 |
| Dimeo 2012 | SOC vs FFT | -0.74132194 | -2.035669 | 0.5442367 | 1 |
| Dimeo 2012 | SOC vs LICT | -0.75095047 | -2.361223 | 0.9207249 | 1 |
| Dimeo 2012 | SOC vs MICT | -0.7187177 | -1.849966 | 0.3411412 | 1 |
| Dimeo 2012 | SOC vs MIIT | -0.53926095 | -3.457173 | 2.17889 | 1 |
| Dimeo 2012 | SOC vs RT | -1.0108838 | -2.369961 | 0.3822933 | 1 |
| Martin 1990 | SOC vs CT | -2.0729006 | -3.296596 | -0.6737847 | 1 |
| Martin 1990 | SOC vs FFT | -0.57214068 | -2.24132 | 0.8941205 | 1 |
| Martin 1990 | SOC vs HICT | -0.66166429 | -2.440843 | 0.3633541 | 1 |
| Martin 1990 | SOC vs HIIT | -0.73892269 | -2.667603 | 0.9858482 | 1 |
| Martin 1990 | SOC vs FFT | -0.74133688 | -2.034222 | 0.5398011 | 1 |
| Martin 1990 | SOC vs LICT | -0.75095671 | -2.359373 | 0.9138103 | 1 |
| Martin 1990 | SOC vs MICT | -0.72443917 | -1.847616 | 0.3252649 | 1 |
| Martin 1990 | SOC vs MIIT | -0.63702793 | -2.828336 | 1.354131 | 1 |
| Martin 1990 | SOC vs RT | -1.0121131 | -2.369494 | 0.3783823 | 1 |
| Abrahin 2022 | SOC vs CT | -2.1113805 | -3.373815 | -0.7016727 | 1 |
| Abrahin 2022 | SOC vs FFT | -0.57119084 | -2.297152 | 0.932363 | 1 |
| Abrahin 2022 | SOC vs HICT | -0.68355629 | -2.310358 | 0.0768412 | 1 |
| Abrahin 2022 | SOC vs HIIT | -0.73899623 | -2.727766 | 1.028643 | 1 |
| Abrahin 2022 | SOC vs FFT | -0.75180367 | -2.086406 | 0.5572324 | 1 |
| Abrahin 2022 | SOC vs LICT | -0.75245692 | -2.411555 | 0.9484959 | 1 |
| Abrahin 2022 | SOC vs MICT | -0.72100209 | -1.953427 | 0.3507973 | 1 |
| Abrahin 2022 | SOC vs MIIT | -0.63608327 | -2.900074 | 1.387062 | 1 |
| Abrahin 2022 | SOC vs RT | -1.1090588 | -2.661186 | 0.4188077 | 1 |
| Herawati 2025 | SOC vs CT | -2.0706903 | -3.28559 | -0.6989037 | 1 |
| Herawati 2025 | SOC vs FFT | -0.57244795 | -2.232146 | 0.8718066 | 1 |
| Herawati 2025 | SOC vs HICT | -0.67846432 | -2.24651 | 0.0363283 | 1 |
| Herawati 2025 | SOC vs HIIT | -0.42046884 | -2.771212 | 1.856922 | 1 |
| Herawati 2025 | SOC vs FFT | -0.74106643 | -2.026836 | 0.5219565 | 1 |
| Herawati 2025 | SOC vs LICT | -0.75093434 | -2.350479 | 0.8923283 | 1 |
| Herawati 2025 | SOC vs MICT | -0.72559687 | -1.840152 | 0.2939623 | 1 |
| Herawati 2025 | SOC vs MIIT | -0.63735277 | -2.816393 | 1.320867 | 1 |
| Herawati 2025 | SOC vs RT | -1.0118828 | -2.361425 | 0.3553834 | 1 |
| López-Ruiz 2025 | SOC vs CT | -0.7697298 | -1.250732 | -0.1783252 | 1 |
| López-Ruiz 2025 | SOC vs FFT | -0.55524661 | -1.130875 | 0.189519 | 1 |
| López-Ruiz 2025 | SOC vs HICT | -0.44936625 | -1.060501 | -0.3625229 | 1 |
| López-Ruiz 2025 | SOC vs HIIT | -0.73139476 | -1.462415 | 0.2275079 | 1 |
| López-Ruiz 2025 | SOC vs FFT | -0.57786068 | -1.058788 | 0.1096515 | 1 |
| López-Ruiz 2025 | SOC vs LICT | -0.73333298 | -1.319104 | 0.2601346 | 1 |
| López-Ruiz 2025 | SOC vs MICT | -0.6217918 | -1.069657 | -0.0557919 | 1 |
| López-Ruiz 2025 | SOC vs MIIT | -0.60158585 | -1.388837 | 0.4656185 | 1 |
| López-Ruiz 2025 | SOC vs RT | -0.68260301 | -1.206967 | 0.0945677 | 1 |
| Lim 2025 | SOC vs CT | -2.0713727 | -3.289095 | -0.689656 | 1 |
| Lim 2025 | SOC vs FFT | -0.5723654 | -2.236736 | 0.8991982 | 1 |
| Lim 2025 | SOC vs HICT | -0.67850087 | -2.250782 | 0.0595432 | 1 |
| Lim 2025 | SOC vs HIIT | -0.7389159 | -2.662319 | 0.9915258 | 1 |
| Lim 2025 | SOC vs FFT | -0.72484784 | -2.017166 | 0.558818 | 1 |
| Lim 2025 | SOC vs LICT | -0.58467198 | -2.419862 | 1.255538 | 1 |
| Lim 2025 | SOC vs MICT | -0.72530031 | -1.842911 | 0.3120439 | 1 |
| Lim 2025 | SOC vs MIIT | -0.63727149 | -2.822373 | 1.356487 | 1 |
| Lim 2025 | SOC vs RT | -1.0107729 | -2.36398 | 0.375589 | 1 |
| Pinto 2025 | SOC vs CT | -2.0805437 | -3.301279 | -0.6944695 | 1 |
| Pinto 2025 | SOC vs FFT | -0.57312888 | -2.241265 | 0.9021803 | 1 |
| Pinto 2025 | SOC vs HICT | -0.68027862 | -2.255992 | 0.0607182 | 1 |
| Pinto 2025 | SOC vs HIIT | -0.73892134 | -2.666543 | 0.9948608 | 1 |
| Pinto 2025 | SOC vs FFT | -0.81917228 | -2.222263 | 0.5696882 | 1 |
| Pinto 2025 | SOC vs LICT | -0.76288119 | -2.372585 | 0.913137 | 1 |
| Pinto 2025 | SOC vs MICT | -0.72835643 | -1.848549 | 0.3114268 | 1 |
| Pinto 2025 | SOC vs MIIT | -0.63815309 | -2.828138 | 1.359593 | 1 |
| Pinto 2025 | SOC vs I | -1.0204669 | -2.377658 | 0.3706435 | 1 |
| Taylor 2018 | SOC vs CT | -2.0289861 | -3.218327 | -0.6839963 | 1 |
| Taylor 2018 | SOC vs FFT | -0.56991149 | -2.184381 | 0.9004212 | 1 |
| Taylor 2018 | SOC vs HICT | -0.67101172 | -2.198126 | 0.0615771 | 1 |
| Taylor 2018 | SOC vs HIIT | -0.73884027 | -2.606266 | 0.9928935 | 1 |
| Taylor 2018 | SOC vs FFT | -0.46834627 | -1.82476 | 0.7038958 | 1 |
| Taylor 2018 | SOC vs LICT | -0.70926896 | -2.268081 | 0.9323003 | 1 |
| Taylor 2018 | SOC vs MICT | -0.71521586 | -1.800361 | 0.3148686 | 1 |
| Taylor 2018 | SOC vs MIIT | -0.63432758 | -2.754706 | 1.358631 | 1 |
| Taylor 2018 | SOC vs RT | -0.9801028 | -2.295534 | 0.3829912 | 1 |
| Izadi 2017 | SOC vs CT | -2.0752143 | -3.296424 | -0.6924591 | 1 |
| Izadi 2017 | SOC vs FFT | -0.57254864 | -2.24283 | 0.89391 | 1 |
| Izadi 2017 | SOC vs HICT | -0.67915171 | -2.256789 | 0.0548489 | 1 |
| Izadi 2017 | SOC vs HIIT | -0.68552479 | -3.044376 | 1.632287 | 1 |
| Izadi 2017 | SOC vs FFT | -0.74158662 | -2.035351 | 0.5379213 | 1 |
| Izadi 2017 | SOC vs LICT | -0.75099253 | -2.360552 | 0.9133492 | 1 |
| Izadi 2017 | SOC vs MICT | -0.72606684 | -1.847536 | 0.3081492 | 1 |
| Izadi 2017 | SOC vs MIIT | -0.63749678 | -2.830267 | 1.349493 | 1 |
| Izadi 2017 | SOC vs RT | -1.0130389 | -2.370816 | 0.370483 | 1 |
| Hou 2023 | SOC vs CT | -2.0975981 | -3.358962 | -0.669809 | 1 |
| Hou 2023 | SOC vs FFT | -0.57261106 | -2.301925 | 0.9370227 | 1 |
| Hou 2023 | SOC vs HICT | -0.68229883 | -2.312143 | 0.0956368 | 1 |
| Hou 2023 | SOC vs HIIT | -0.73900016 | -2.731142 | 1.033863 | 1 |
| Hou 2023 | SOC vs FFT | -0.74412984 | -2.079845 | 0.5698543 | 1 |
| Hou 2023 | SOC vs LICT | -0.75127558 | -2.413194 | 0.9544685 | 1 |
| Hou 2023 | SOC vs MICT | -0.72666139 | -1.968789 | 0.4295382 | 1 |
| Hou 2023 | SOC vs MIIT | -0.76523787 | -4.198917 | 2.046841 | 1 |
| Hou 2023 | SOC vs RT | -1.0183332 | -2.425956 | 0.4109561 | 1 |
| Alzahrani 2023 | SOC vs CT | -2.073989 | -3.291806 | -0.6904503 | 1 |
| Alzahrani 2023 | SOC vs FFT | -0.57261026 | -2.237199 | 0.9023073 | 1 |
| Alzahrani 2023 | SOC vs HICT | -0.67902666 | -2.251504 | 0.0617885 | 1 |
| Alzahrani 2023 | SOC vs HIIT | -0.73891622 | -2.662564 | 0.9950027 | 1 |
| Alzahrani 2023 | SOC vs FFT | -0.7562659 | -2.048623 | 0.5394392 | 1 |
| Alzahrani 2023 | SOC vs LICT | -0.90217848 | -2.728177 | 1.029453 | 1 |
| Alzahrani 2023 | SOC vs MICT | -0.72627385 | -1.844027 | 0.3133235 | 1 |
| Alzahrani 2023 | SOC vs MIIT | -0.63755151 | -2.822936 | 1.360308 | 1 |
| Alzahrani 2023 | SOC vs RT | -1.0138938 | -2.367268 | 0.3755854 | 1 |
| Gambassi 2024 | SOC vs CT | -2.2326808 | -3.535768 | -0.7826232 | 1 |
| Gambassi 2024 | SOC vs FFT | -0.58148241 | -2.24288 | 0.8899018 | 1 |
| Gambassi 2024 | SOC vs HICT | -0.70762947 | -2.279268 | 0.0236633 | 1 |
| Gambassi 2024 | SOC vs HIIT | -0.73891129 | -2.658756 | 0.9811323 | 1 |
| Gambassi 2024 | SOC vs FFT | -0.75632593 | -2.044 | 0.5208515 | 1 |
| Gambassi 2024 | SOC vs LICT | -0.75326858 | -2.354248 | 0.9072877 | 1 |
| Gambassi 2024 | SOC vs MICT | -0.76147407 | -1.881692 | 0.2714919 | 1 |
| Gambassi 2024 | SOC vs MIIT | -0.64766693 | -2.828853 | 1.33345 | 1 |
| Gambassi 2024 | SOC vs RT | -1.0573655 | -2.414448 | 0.3276635 | 1 |
| Nemoto 2021 | SOC vs CT | -2.0818615 | -3.300275 | -0.6939844 | 1 |
| Nemoto 2021 | SOC vs FFT | -0.57337668 | -2.237677 | 0.9030387 | 1 |
| Nemoto 2021 | SOC vs HICT | -0.68062916 | -2.252865 | 0.0614861 | 1 |
| Nemoto 2021 | SOC vs HIIT | -0.73891579 | -2.662234 | 0.9958209 | 1 |
| Nemoto 2021 | SOC vs FFT | -0.85574702 | -2.257617 | 0.5753731 | 1 |
| Nemoto 2021 | SOC vs LICT | -0.768491 | -2.374601 | 0.914409 | 1 |
| Nemoto 2021 | SOC vs MICT | -0.72931472 | -1.847002 | 0.3120711 | 1 |
| Nemoto 2021 | SOC vs MIIT | -0.63842534 | -2.823435 | 1.360732 | 1 |
| Nemoto 2021 | SOC vs RT | -1.0236742 | -2.37789 | 0.3715186 | 1 |
| Ma 2018 | SOC vs CT | -2.0784023 | -3.298742 | -0.6890466 | 1 |
| Ma 2018 | SOC vs FFT | -0.71302073 | -2.648907 | 1.146424 | 1 |
| Ma 2018 | SOC vs HICT | -0.68187847 | -2.257863 | 0.0642851 | 1 |
| Ma 2018 | SOC vs HIIT | -0.73892161 | -2.666756 | 0.9981662 | 1 |
| Ma 2018 | SOC vs FFT | -0.74214209 | -2.034432 | 0.5460452 | 1 |
| Ma 2018 | SOC vs LICT | -0.75108157 | -2.358784 | 0.9240292 | 1 |
| Ma 2018 | SOC vs MICT | -0.74192494 | -1.867639 | 0.3153718 | 1 |
| Ma 2018 | SOC vs MIIT | -0.64205331 | -2.832511 | 1.36404 | 1 |
| Ma 2018 | SOC vs RT | -1.0176577 | -2.374325 | 0.3781905 | 1 |
| Chauhan 2017 | SOC vs CT | -2.0782797 | -3.298688 | -0.6891928 | 1 |
| Chauhan 2017 | SOC vs FFT | -0.70706519 | -2.640873 | 1.059817 | 1 |
| Chauhan 2017 | SOC vs HICT | -0.68176475 | -2.25785 | 0.0638843 | 1 |
| Chauhan 2017 | SOC vs HIIT | -0.73892177 | -2.666882 | 0.9976333 | 1 |
| Chauhan 2017 | SOC vs FFT | -0.74211998 | -2.034495 | 0.5457002 | 1 |
| Chauhan 2017 | SOC vs LICT | -0.75107795 | -2.358887 | 0.9235759 | 1 |
| Chauhan 2017 | SOC vs MICT | -0.74125382 | -1.866995 | 0.3150651 | 1 |
| Chauhan 2017 | SOC vs MIIT | -0.6418605 | -2.832463 | 1.363423 | 1 |
| Chauhan 2017 | SOC vs RT | -1.0174651 | -2.374217 | 0.3778629 | 1 |
| Dhungana 2021 | SOC vs CT | -2.0706591 | -3.291161 | -0.6896073 | 1 |
| Dhungana 2021 | SOC vs FFT | -0.44031248 | -2.376206 | 1.205809 | 1 |
| Dhungana 2021 | SOC vs HICT | -0.67634927 | -2.252472 | 0.0627431 | 1 |
| Dhungana 2021 | SOC vs HIIT | -0.73892182 | -2.666923 | 0.9961163 | 1 |
| Dhungana 2021 | SOC vs FFT | -0.74088516 | -2.033288 | 0.5447181 | 1 |
| Dhungana 2021 | SOC vs LICT | -0.75088873 | -2.358732 | 0.9222854 | 1 |
| Dhungana 2021 | SOC vs MICT | -0.71098113 | -1.836807 | 0.3141918 | 1 |
| Dhungana 2021 | SOC vs MIIT | -0.63315911 | -2.823811 | 1.361665 | 1 |
| Dhungana 2021 | SOC vs RT | -1.0082948 | -2.365091 | 0.3769304 | 1 |
| Georgiades 2000 | SOC vs CT | -2.3294948 | -3.681667 | -0.6875051 | 1 |
| Georgiades 2000 | SOC vs FFT | -0.58490155 | -2.285094 | 0.9372852 | 1 |
| Georgiades 2000 | SOC vs HICT | -0.64083803 | -2.537164 | 0.430457 | 1 |
| Georgiades 2000 | SOC vs HIIT | -0.73896597 | -2.702336 | 1.034157 | 1 |
| Georgiades 2000 | SOC vs FFT | -0.76568189 | -2.082868 | 0.5609624 | 1 |
| Georgiades 2000 | SOC vs LICT | -0.7546256 | -2.392307 | 0.9532421 | 1 |
| Georgiades 2000 | SOC vs MICT | -0.77531528 | -1.921291 | 0.3247018 | 1 |
| Georgiades 2000 | SOC vs MIIT | -0.65167357 | -2.883186 | 1.401292 | 1 |
| Georgiades 2000 | SOC vs RT | -1.0827347 | -2.471193 | 0.3800814 | 1 |
| Hagberg 1989 | SOC vs CT | -2.1091143 | -3.366382 | -0.6896448 | 1 |
| Hagberg 1989 | SOC vs FFT | -0.58053548 | -2.303883 | 0.933305 | 1 |
| Hagberg 1989 | SOC vs HICT | -0.76012949 | -2.663788 | 0.2813746 | 1 |
| Hagberg 1989 | SOC vs HIIT | -0.73899236 | -2.724457 | 1.029699 | 1 |
| Hagberg 1989 | SOC vs FFT | -0.74568495 | -2.076881 | 0.5643978 | 1 |
| Hagberg 1989 | SOC vs LICT | -0.75152486 | -2.407815 | 0.9505101 | 1 |
| Hagberg 1989 | SOC vs MICT | -0.75810181 | -1.995849 | 0.3617414 | 1 |
| Hagberg 1989 | SOC vs MIIT | -0.64674104 | -2.90724 | 1.389927 | 1 |
| Hagberg 1989 | SOC vs RT | -1.0286623 | -2.430933 | 0.3880105 | 1 |
| Cahu Rodrigues 2019 | SOC vs CT | -2.0656826 | -3.284253 | -0.6838914 | 1 |
| Cahu Rodrigues 2019 | SOC vs FFT | -0.57181871 | -2.236176 | 0.8989998 | 1 |
| Cahu Rodrigues 2019 | SOC vs HICT | -0.6773476 | -2.249638 | 0.0605031 | 1 |
| Cahu Rodrigues 2019 | SOC vs HIIT | -0.73891587 | -2.662298 | 0.9913039 | 1 |
| Cahu Rodrigues 2019 | SOC vs FFT | -0.65415726 | -2.051307 | 0.707544 | 1 |
| Cahu Rodrigues 2019 | SOC vs LICT | -0.73761264 | -2.343678 | 0.9319856 | 1 |
| Cahu Rodrigues 2019 | SOC vs MICT | -0.72312977 | -1.84085 | 0.3141884 | 1 |
| Cahu Rodrigues 2019 | SOC vs MIIT | -0.63664757 | -2.82173 | 1.356857 | 1 |
| Cahu Rodrigues 2019 | SOC vs RT | -1.0037993 | -2.358026 | 0.3826925 | 1 |
| Pagonas 2017 | SOC vs CT | -2.1058264 | -3.347515 | -0.6898618 | 1 |
| Pagonas 2017 | SOC vs FFT | -0.57456911 | -2.274756 | 0.9307526 | 1 |
| Pagonas 2017 | SOC vs HICT | -0.68471789 | -2.289525 | 0.0837578 | 1 |
| Pagonas 2017 | SOC vs HIIT | -0.73896625 | -2.702565 | 1.02684 | 1 |
| Pagonas 2017 | SOC vs FFT | -0.96850967 | -2.427942 | 0.5537817 | 1 |
| Pagonas 2017 | SOC vs LICT | -0.65050277 | -2.602366 | 1.221208 | 1 |
| Pagonas 2017 | SOC vs MICT | -0.73428245 | -1.875475 | 0.3277872 | 1 |
| Pagonas 2017 | SOC vs MIIT | -0.63988204 | -2.871451 | 1.396037 | 1 |
| Pagonas 2017 | SOC vs RT | -1.038464 | -2.421023 | 0.3844067 | 1 |

Network Grade Classification

Table S11: CINeMA's Assessment of the Credibility of SBP Evidence

| Comparison | Number of studies | Within-study bias | Reporting bias | Indirectness | Imprecision | Heterogeneity | Incoherence | Confidence rating | Reason(s) for downgrading |
| --- | --- | --- | --- | --- | --- | --- | --- | --- | --- |
| CT:HICT | 1 | No concerns | Low risk | No concerns | No concerns | No concerns | No concerns | High | [] |
| CT:IFT | 1 | No concerns | Low risk | No concerns | Major concerns | No concerns | No concerns | Low | ["Imprecision"] |
| CT:MICT | 3 | No concerns | Low risk | No concerns | Major concerns | No concerns | No concerns | Low | ["Imprecision"] |
| CT:RT | 3 | No concerns | Low risk | No concerns | Major concerns | No concerns | No concerns | Low | ["Imprecision"] |
| CT:SOC | 7 | No concerns | Low risk | No concerns | No concerns | No concerns | No concerns | High | [] |
| FFT:SOC | 4 | No concerns | Low risk | No concerns | No concerns | No concerns | Major concerns | Low | ["Incoherence"] |
| HICT:MICT | 1 | Some concerns | Low risk | No concerns | Major concerns | No concerns | No concerns | High | ["Within-study bias","Imprecision"] |
| HICT:SOC | 4 | No concerns | Low risk | No concerns | No concerns | No concerns | No concerns | High | [] |
| HIIT:SOC | 3 | No concerns | Low risk | No concerns | Some concerns | Some concerns | Major concerns | Very low | ["Imprecision","Heterogeneity","Incoherence"] |
| IFT:LICT | 1 | Some concerns | Low risk | No concerns | Major concerns | No concerns | No concerns | Very low | ["Within-study bias","Imprecision"] |
| IFT:RT | 1 | Some concerns | Low risk | No concerns | Major concerns | No concerns | No concerns | Very low | ["Within-study bias","Imprecision"] |
| IFT:SOC | 6 | No concerns | Low risk | No concerns | No concerns | No concerns | No concerns | High | [] |
| LICT:SOC | 4 | No concerns | Low risk | No concerns | Some concerns | No concerns | No concerns | Moderate | ["Imprecision"] |
| MICT:MIIT | 1 | Some concerns | Low risk | No concerns | Major concerns | No concerns | No concerns | Very low | ["Within-study bias","Imprecision"] |
| MICT:RT | 3 | No concerns | Low risk | No concerns | Major concerns | No concerns | No concerns | Low | ["Imprecision"] |
| MICT:SOC | 7 | No concerns | Low risk | No concerns | No concerns | No concerns | No concerns | High | [] |
| MIIT:SOC | 2 | No concerns | Low risk | No concerns | No concerns | No concerns | No concerns | High | [] |
| RT:SOC | 5 | No concerns | Low risk | No concerns | No concerns | Some concerns | No concerns | Moderate | ["Heterogeneity"] |
| CT:FFT | 0 | No concerns | Low risk | No concerns | Major concerns | No concerns | Major concerns | Very low | ["Imprecision","Incoherence"] |
| CT:HIIT | 0 | No concerns | Low risk | No concerns | Major concerns | No concerns | Major concerns | Very low | ["Imprecision","Incoherence"] |
| CT:LICT | 0 | No concerns | Low risk | No concerns | Major concerns | No concerns | Major concerns | Very low | ["Imprecision","Incoherence"] |
| CT:MIIT | 0 | No concerns | Low risk | No concerns | Major concerns | No concerns | No concerns | Low | ["Imprecision"] |
| FFT:HICT | 0 | Some concerns | Low risk | No concerns | Major concerns | No concerns | Major concerns | Very low | ["Within-study bias","Imprecision","Incoherence"] |
| FFT:HIIT | 0 | Some concerns | Low risk | No concerns | Major concerns | No concerns | Major concerns | Very low | ["Within-study bias","Imprecision","Incoherence"] |
| FFT:IFT | 0 | Some concerns | Low risk | No concerns | Major concerns | No concerns | Major concerns | Very low | ["Within-study bias","Imprecision","Incoherence"] |
| FFT:LICT | 0 | Some concerns | Low risk | No concerns | Major concerns | No concerns | Major concerns | Very low | ["Within-study bias","Imprecision","Incoherence"] |
| FFT:MICT | 0 | Some concerns | Low risk | No concerns | Major concerns | No concerns | Major concerns | Very low | ["Within-study bias","Imprecision","Incoherence"] |
| FFT:MIIT | 0 | Some concerns | Low risk | No concerns | Major concerns | No concerns | Major concerns | Very low | ["Within-study bias","Imprecision","Incoherence"] |
| FFT:RT | 0 | Some concerns | Low risk | No concerns | Major concerns | No concerns | Major concerns | Very low | ["Within-study bias","Imprecision","Incoherence"] |
| HICT:HIIT | 0 | Major concerns | Low risk | No concerns | Major concerns | No concerns | Major concerns | Very low | ["Within-study bias","Imprecision","Incoherence"] |
| HICT:IFT | 0 | Some concerns | Low risk | No concerns | Major concerns | No concerns | Major concerns | Very low | ["Within-study bias","Imprecision","Incoherence"] |
| HICT:LICT | 0 | Some concerns | Low risk | No concerns | Major concerns | No concerns | Major concerns | Very low | ["Within-study bias","Imprecision","Incoherence"] |
| HICT:MIIT | 0 | Some concerns | Low risk | No concerns | Major concerns | No concerns | Major concerns | Very low | ["Within-study bias","Imprecision","Incoherence"] |
| HICT:RT | 0 | Some concerns | Low risk | No concerns | Major concerns | No concerns | Major concerns | Very low | ["Within-study bias","Imprecision","Incoherence"] |
| HIIT:IFT | 0 | Some concerns | Low risk | No concerns | Major concerns | No concerns | Major concerns | Very low | ["Within-study bias","Imprecision","Incoherence"] |
| HIIT:LICT | 0 | Some concerns | Low risk | No concerns | Major concerns | No concerns | Major concerns | Very low | ["Within-study bias","Imprecision","Incoherence"] |
| HIIT:MICT | 0 | Some concerns | Low risk | No concerns | Major concerns | No concerns | Major concerns | Very low | ["Within-study bias","Imprecision","Incoherence"] |
| HIIT:MIIT | 0 | Some concerns | Low risk | No concerns | Major concerns | No concerns | Major concerns | Very low | ["Within-study bias","Imprecision","Incoherence"] |
| HIIT:RT | 0 | Some concerns | Low risk | No concerns | Major concerns | No concerns | Major concerns | Very low | ["Within-study bias","Imprecision","Incoherence"] |
| IFT:MICT | 0 | Some concerns | Low risk | No concerns | Major concerns | No concerns | Major concerns | Very low | ["Within-study bias","Imprecision","Incoherence"] |
| IFT:MIIT | 0 | Some concerns | Low risk | No concerns | Major concerns | No concerns | Major concerns | Very low | ["Within-study bias","Imprecision","Incoherence"] |
| LICT:MICT | 0 | Some concerns | Low risk | No concerns | Major concerns | No concerns | Major concerns | Very low | ["Within-study bias","Imprecision","Incoherence"] |
| LICT:MIIT | 0 | Some concerns | Low risk | No concerns | Major concerns | No concerns | Major concerns | Very low | ["Within-study bias","Imprecision","Incoherence"] |
| LICT:RT | 0 | Some concerns | Low risk | No concerns | Major concerns | No concerns | Major concerns | Very low | ["Within-study bias","Imprecision","Incoherence"] |
| MIIT:RT | 0 | No concerns | Low risk | No concerns | No concerns | No concerns | No concerns | High | [] |

Table S12: CINeMA's assessment of the credibility of DBP evidence

| Comparison | Number of studies | Within-study bias | Reporting bias | Indirectness | Imprecision | Heterogeneity | Incoherence | Confidence rating | Reason(s) for downgrading |
| --- | --- | --- | --- | --- | --- | --- | --- | --- | --- |
| CT:HICT | 1 | No concerns | Low risk | No concerns | No concerns | No concerns | No concerns | High | [] |
| CT:IFT | 1 | No concerns | Low risk | No concerns | No concerns | No concerns | No concerns | High | [] |
| CT:MICT | 3 | No concerns | Low risk | No concerns | No concerns | No concerns | No concerns | High | [] |
| CT:RT | 3 | No concerns | Low risk | No concerns | No concerns | No concerns | No concerns | High | [] |
| CT:SOC | 7 | No concerns | Low risk | No concerns | No concerns | Major concerns | No concerns | Low | ["Heterogeneity"] |
| FFT:SOC | 4 | No concerns | Low risk | No concerns | No concerns | No concerns | No concerns | Low | [] |
| HICT:MICT | 1 | Some concerns | Low risk | No concerns | Major concerns | No concerns | No concerns | Very low | ["Within-study bias","Imprecision"] |
| HICT:SOC | 4 | No concerns | Low risk | No concerns | No concerns | Major concerns | No concerns | Low | ["Heterogeneity"] |
| HIIT:SOC | 3 | No concerns | Low risk | No concerns | No concerns | No concerns | No concerns | High | [] |
| IFT:LICT | 1 | Some concerns | Low risk | No concerns | Major concerns | No concerns | No concerns | Very low | ["Within-study bias","Imprecision"] |
| IFT:RT | 1 | Some concerns | Low risk | No concerns | Major concerns | No concerns | No concerns | Very low | ["Within-study bias","Imprecision"] |
| IFT:SOC | 6 | No concerns | Low risk | No concerns | No concerns | Some concerns | No concerns | Moderate | ["Heterogeneity"] |
| LICT:SOC | 4 | No concerns | Low risk | No concerns | No concerns | No concerns | No concerns | High | [] |
| MICT:MIIT | 1 | Some concerns | Low risk | No concerns | Major concerns | No concerns | No concerns | Very low | ["Within-study bias","Imprecision"] |
| MICT:RT | 3 | Some concerns | Low risk | No concerns | Major concerns | No concerns | No concerns | Very low | ["Within-study bias","Imprecision"] |
| MICT:SOC | 7 | No concerns | Low risk | No concerns | No concerns | No concerns | No concerns | High | [] |
| MIIT:SOC | 2 | No concerns | Low risk | No concerns | No concerns | No concerns | No concerns | High | [] |
| RT:SOC | 5 | No concerns | Low risk | No concerns | No concerns | Some concerns | No concerns | Moderate | ["Heterogeneity"] |
| CT:FFT | 0 | No concerns | Low risk | No concerns | No concerns | No concerns | Major concerns | Low | ["Incoherence"] |
| CT:HIIT | 0 | No concerns | Low risk | No concerns | No concerns | No concerns | Major concerns | Low | ["Incoherence"] |
| CT:LICT | 0 | No concerns | Low risk | No concerns | No concerns | No concerns | Major concerns | Low | ["Incoherence"] |
| CT:MIIT | 0 | No concerns | Low risk | No concerns | No concerns | No concerns | Major concerns | Low | ["Incoherence"] |
| FFT:HICT | 0 | Some concerns | Low risk | No concerns | Major concerns | No concerns | Major concerns | Very low | ["Within-study bias","Imprecision","Incoherence"] |
| FFT:HIIT | 0 | Some concerns | Low risk | No concerns | Major concerns | No concerns | Major concerns | Very low | ["Within-study bias","Imprecision","Incoherence"] |
| FFT:IFT | 0 | Some concerns | Low risk | No concerns | Major concerns | No concerns | Major concerns | Very low | ["Within-study bias","Imprecision","Incoherence"] |
| FFT:LICT | 0 | Some concerns | Low risk | No concerns | Major concerns | No concerns | Major concerns | Very low | ["Within-study bias","Imprecision","Incoherence"] |
| FFT:MICT | 0 | Some concerns | Low risk | No concerns | No concerns | No concerns | Major concerns | Very low | ["Within-study bias","Incoherence"] |
| FFT:MIIT | 0 | Some concerns | Low risk | No concerns | No concerns | No concerns | Major concerns | Very low | ["Within-study bias","Incoherence"] |
| FFT:RT | 0 | Some concerns | Low risk | No concerns | No concerns | No concerns | Major concerns | Very low | ["Within-study bias","Incoherence"] |
| HICT:HIIT | 0 | Major concerns | Low risk | No concerns | Some concerns | No concerns | Major concerns | Very low | ["Within-study bias","Imprecision","Incoherence"] |
| HICT:IFT | 0 | Some concerns | Low risk | No concerns | No concerns | No concerns | Major concerns | Very low | ["Within-study bias","Incoherence"] |
| HICT:LICT | 0 | Some concerns | Low risk | No concerns | Some concerns | No concerns | Major concerns | Very low | ["Within-study bias","Imprecision","Incoherence"] |
| HICT:MIIT | 0 | Some concerns | Low risk | No concerns | Major concerns | No concerns | Major concerns | Very low | ["Within-study bias","Imprecision","Incoherence"] |
| HICT:RT | 0 | Some concerns | Low risk | No concerns | No concerns | No concerns | Some concerns | Low | ["Within-study bias","Incoherence"] |
| HIIT:IFT | 0 | Some concerns | Low risk | No concerns | No concerns | No concerns | Some concerns | Low | ["Within-study bias","Incoherence"] |
| HIIT:LICT | 0 | Some concerns | Low risk | No concerns | No concerns | No concerns | Some concerns | Low | ["Within-study bias","Incoherence"] |
| HIIT:MICT | 0 | Some concerns | Low risk | No concerns | No concerns | No concerns | Some concerns | Low | ["Within-study bias","Incoherence"] |
| HIIT:MIIT | 0 | Some concerns | Low risk | No concerns | No concerns | No concerns | Some concerns | Low | ["Within-study bias","Incoherence"] |
| HIIT:RT | 0 | Some concerns | Low risk | No concerns | No concerns | No concerns | Some concerns | Low | ["Within-study bias","Incoherence"] |
| IFT:MICT | 0 | Some concerns | Low risk | No concerns | Major concerns | No concerns | No concerns | Very low | ["Within-study bias","Imprecision"] |
| IFT:MIIT | 0 | Some concerns | Low risk | No concerns | Major concerns | No concerns | Major concerns | Very low | ["Within-study bias","Imprecision","Incoherence"] |
| LICT:MICT | 0 | Some concerns | Low risk | No concerns | No concerns | No concerns | Some concerns | Low | ["Within-study bias","Incoherence"] |
| LICT:MIIT | 0 | Some concerns | Low risk | No concerns | No concerns | No concerns | Major concerns | Very low | ["Within-study bias","Incoherence"] |
| LICT:RT | 0 | Some concerns | Low risk | No concerns | No concerns | No concerns | Major concerns | Very low | ["Within-study bias","Incoherence"] |
| MIIT:RT | 0 | No concerns | Low risk | No concerns | No concerns | No concerns | No concerns | High | [] |
